# Antibacterial Polyketides from Antarctica Sponge-Derived Fungus *Penicillium* sp. HDN151272

**DOI:** 10.3390/md18020071

**Published:** 2020-01-23

**Authors:** Mudassir Shah, Chunxiao Sun, Zichao Sun, Guojian Zhang, Qian Che, Qianqun Gu, Tianjiao Zhu, Dehai Li

**Affiliations:** 1 Key Laboratory of Marine Drugs, Chinese Ministry of Education, School of Medicine and Pharmacy, Ocean University of China, Qingdao 266003, China; s84mudassir@gmail.com (M.S.); sunchunxiao93@163.com (C.S.); 171774170@163.com (Z.S.); zhangguojian@ouc.edu.cn (G.Z.); cheqian064@ouc.edu.cn (Q.C.); guqianq@ouc.edu.cn (Q.G.); 2 Laboratory for Marine Drugs and Bioproducts, Pilot National Laboratory for Marine Science and Technology, Qingdao 266237, China; 3 Open Studio for Druggability Research of Marine Natural Products, Pilot National Laboratory for Marine Science and Technology, Qingdao 266237, China

**Keywords:** Antarctica sponge-derived fungus, *Penicillium* sp., polyketides, antibacterial activity

## Abstract

Three new polyketides, ketidocillinones A–C (**1**–**3**), were discovered from the extract of an Antarctica sponge-derived fungus *Penicillium* sp. HDN151272. All the structures were deduced by spectroscopic data, including NMR and HRESIMS. The absolute configuration of compound **3** was established by using ECD calculation. Compounds **1**−**3** can be slowly oxidized to quinone form when exposed to air. Ketidocillinones B and C (**2** and **3**) exhibited potent antibacterial activity against *Pseudomonas aeurigenosa*, *Mycobacterium phlei*, and MRCNS (methicillin-resistant coagulase-negative *staphylococci*) with MIC values ranging from 1.56 to 25.00 µg/mL.

## 1. Introduction

Microorganisms that belong to distinctive ecosystems, such as Polar Regions, have productive sources of different chemical skeletons and unusual natural products along with versatile and unparalleled biological potentials [1]. Polar regions, including the arctic, the Antarctic, and other related subregions, are considered the most inaccessible and arduous domain on the planet. To continue existence and endurance under the persistent influence of natural stressors, such as extremely low temperature, deficient of nutritional substrates, lack of metabolite transfers, UV (ultra violet-radiation) and interim concentrated heat during the Antarctic summer, the Antarctica associated microorganisms need a different range of bio-physiological adaptations which are necessary for existence [1,2]. These adaptations are frequently convoyed by manipulations to both gene regulation and metabolic pathways, in turn enlarging the opportunity to search for innovative functional metabolites with pharmaceutical potential [1,3]. The Antarctic-derived microorganisms would have the opportunity to produce novel metabolites with unusual and exclusive structures along with attractive biological activities [4,5,6,7].

**Figure 1:**
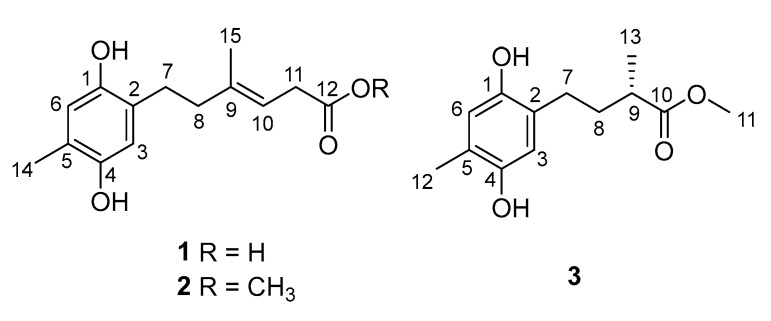
Structures of compounds **1**–**3**.

During our efforts to explore new bioactive molecules from Antarctica sponge-derived microorganisms [8,9,10], a fungal strain *Penicillium* sp. HDN151272, isolated from an unidentified sponge sample collected in the Prydz Bay, was selected for chemical investigation due to its interesting HPLC-UV profile. A chemical investigation of the organic extract of the fungus led to the discovery of three hydroquinone polyketides (Figure 1), namely ketidocillinones A–C (**1**−**3**), which displayed different side chains compared with the previously reported hydroquinone polyketides [11,12]. Compounds **2** and **3** exhibited broad-spectrum antibacterial activity. Herein, we report the details of the isolation, structure elucidation, absolute configuration, and biological activities of the new compounds.

## 2. Results and Discussion

The fungal strain *Penicillium* sp. HDN151272 was cultured at 28 °C for 9 days under shaking condition. The extract (10 g) of the fermentation (30 L) was fractionated by ODS and LH-20 column chromatography and HPLC using ODS, producing compounds **1** (7.0 mg), **2** (6.0 mg), **3** (5.5 mg).

**Figure 2:**
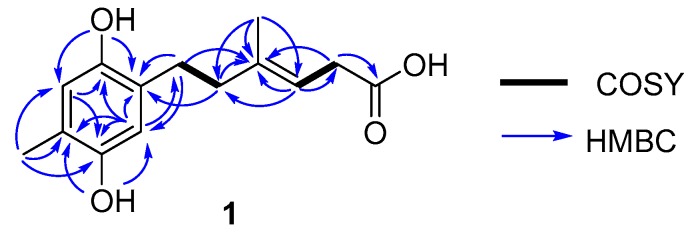
Key COSY and HMBC correlations of **1**.

Compound **1** was isolated as a deep yellowish powder, and the HRESIMS ion detected at *m*/*z* 249.1134 [M − H]^−^ indicated a molecular formula of C_14_H_18_O_4_, accounting for six degrees of unsaturation. ^1^H NMR data (Table 1) displayed resonances for two methyls (*δ*_H_ 1.60, s, H_3_-14; 1.97, s, H_3_-15), three methylenes (*δ*_H_ 2.48, m, H_2_-7; 2.12, m, H_2_-8; 2.93, d, H_2_-11), three olefinic protons (*δ*_H_ 6.44, s, H-3; 6.44, H-6; 5.26, t, H-10), and two phenolic hydroxyls (*δ*_H_ 8.34, s, 1-OH; 8.30, s, 4-OH). The ^13^C NMR as well as DEPT and HSQC spectrum data in dicated 14 carbon resonances assignable to two sp^3^ methyls (*δ*_C_ 16.7, C-14; 16.2, C-15), three sp^3^ methylenes (*δ*_C_ 28.7, C-7; 39.9, C-8; 33.7, C-11), three sp^2^ methines (*δ*_C_ 116.2, C-3; 117.5, C-6; 116.8, C-10), and six quaternary carbons (*δ*_C_ 147.5, C-1; 126.0, C-2; 147.9, C-4; 121.7, C-5; 138.2, C-9; 173.5, C-12). Careful analysis of the ^13^C NMR data of **1** revealed a hydroquinone-type skeleton, which was further verified by the HMBC correlations (Figure 2). Detailed analysis of the COSY spectrum revealed spin systems, including H-7/H-8 and H-10/H-11 (Figure 2). The presence of side chain C-7/C-8/C-9(C-15)/C-10/C-11/C-12 was supported by the HMBC correlations from H-15 to C-8, C-9, and C-10, and from H-11 to C-9, C-10 and C-12. Further HMBC cross peaks from H-7 to C-2 and C-3 and from H-8 to C-2 attached the side chain to C-2. The *E* geometry of the double bond between C-9 and C-10 was inferred based on the NOESY correlations from H-15 to H-11 and from H-10 to H-8. Accordingly, the structure of **1** was established as shown and named as ketidocillinone A.

**Figure 3:**
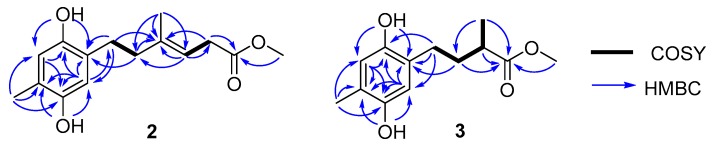
Key COSY and HMBC correlations of compounds **2**–**3**.

Compound **2** was isolated as a deep yellowish powder. The HRESIMS peak at *m*/*z* 263.1289 [M − H]^−^ indicated a molecular formula of C_15_H_20_O_4_, with 14 mass units more than **1**. The 1D NMR data (Table 1) indicated one extra oxygenated methyl group in the structure than **1**. The observed HMBC (Figure 3) correlation from H-13 to C-12 confirmed that compound **2** was a methyl ester of **1**. The geometrical configuration of the double bond between C-9 and C-10 was inferred to be *E* based on the NOESY correlations from H-15 to H-11 and from H-10 to H-8. Thus, the structure of **2** was fully established, as shown and named as ketidocillinone B.

Compound **3**, isolated as a light yellowish powder, was deduced to have a molecular formula of C_13_H_18_O_4_ by HRESIMS. Analysis of the ^1^H and ^13^C NMR (Table 1), as well as the HSQC data of **3**, revealed the presence of three methyls including an oxygenated (*δ*_C_ 51.7, C-11), two methylenes (*δ*_C_ 27.3, C-7; 33.9, C-8), three methines (*δ*_C_ 116.2, C-3; 117.5, C-6; 38.6, C-9), and five nonprotonated sp^2^ carbons (*δ*_C_ 147.5, C-1; 125.4, C-2; 147.9, C-4; 121.9, C-5; 176.7, C-10). The almost identical UV spectra of **1**–**3** suggested they share the same aromatic chromophore, which was further confirmed by the HMBC correlations (Figure 3). The MS and 1D NMR data revealed a shortened side chain in **3**, which was confirmed by COSY correlations of H-7/H-8/H-9/H-13 and HMBC correlations from H-7 to C-9, from H-8 to C-10 and C-13, from H-13 to C-8 and C-10, from H-11 to C-10 (Figure 3). Further HMBC cross peaks from H-7 to C-2 and C-3 and from H-8 to C-2 attached the side chain to C-2. Consequently, the planar structure of **3** was established as shown and named as ketidocillinone C.

**Figure 4:**
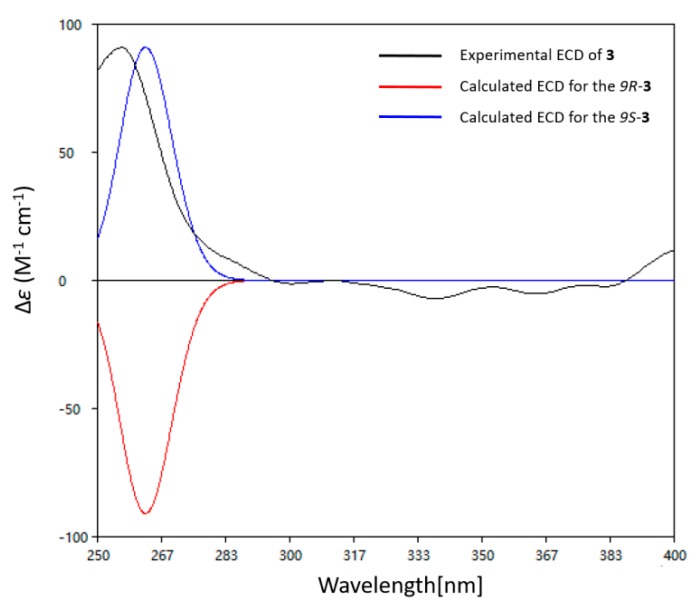
Calculated and experimental ECD spectra of **3** in DMSO.

The absolute configuration of C-9 in compound **3** was deduced by the calculated ECD spectra. The theoretical calculated electronic circular dichroism spectra were performed using TDDFT (time-dependent density functional theory). The optimized conformation of the model was obtained and further used for the ECD calculation at the B3LYP/6-31G (+d) level. The overall pattern of the experimental ECD spectrum was in reasonable agreement with the calculated ECD spectra (Figure 4). Thus, the absolute configuration of **3** was established as 9*S*.

**Figure 5:**
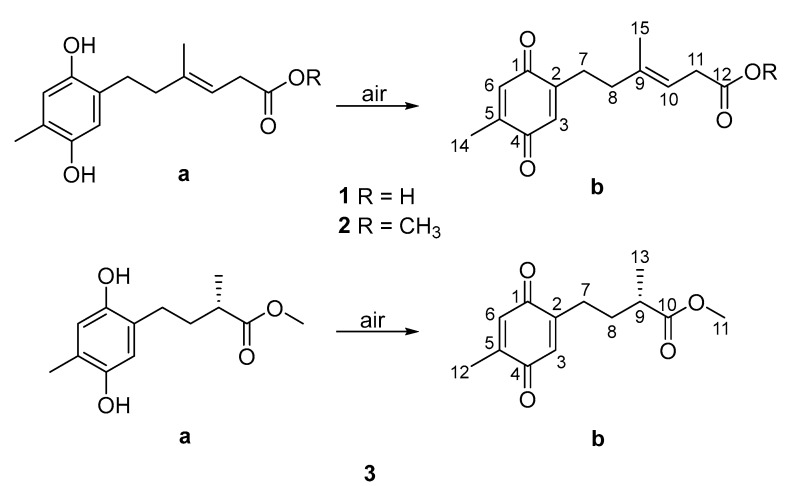
Oxidative transformation of **1**–**3**.

Dihydroquinones can sometimes be converted to quinones by oxidative transformation. For instance, the natural *p*-dihydroquinones [11], and fallahydroquinone [12], isolated from the Western Australian brown alga *Cystophora* sp., and the southern Australian brown alga *Sargassum fallax*, respectively, were discovered to be the results of rapid air mediated oxidation on the (2′*E*)-2-(3′,7′-dimethylocta-2′,6′-dienyl)-6-methyl-2,5-cyclohexadiene-1,4-dione moiety. With regard to compounds ketidocillinones A–C, after exposure to air for about one week, these pure compounds were transformed into mixtures of both enol/keto form structures (Figure 5) with the ratios (enol:keto) 1:1 for **1** (Figure S32), 2:1 for **2** (Figure S39), and 5:4 for **3** (Figure S46), which again proved the non-enzymatic oxidation transformation from dihydroquinones to quinones in natural products. To classify the results, we also collected the NMR data of the mixture of enol/keto forms of **1**–**3** (Table 2 and Table 3, Figures S32–S51).

All the compounds were tested for antimicrobial activity. Compound **2** (a mixture of **2a** and **2b**) exhibited inhibitory activity against *Mycobactrium phlei*, *Pseudomonas aeruginosa*, MRCNS and *Bacillus cereus* with MIC ranging from 1.56 to 12.50 µg/mL, respectively, while compound **1** (mixture of **1a** and **1b**) was inactive (MIC > 50 µg/mL), which indicated that the methoxy group played a crucial role for the activity. Compound **3** (a mixture of **3a** and **3b**) also showed antibacterial activity against MRCNS, *Mycobactrium phlei*, *Pseudomonas aeruginosa*, *Vibrio parahemolyticus*, and *Bacillus subtilis* with MIC ranging from 6.25 to 25.00 µg/mL (Table 4).

## 3. Materials and Methods

### 3.1. General Experimental Procedures

Optical rotations were recorded on a JASCO P-1020 (JASCO Corporation, Tokyo, Japan) digital polarimeter. UV spectra were obtained on HITACHI Chromaster 5430 (HITACHI Corporation, Tokyo, Japan), while the ECD spectra were obtained on JASCO J-815 spectropolarimeter (JASCO Corporation, Tokyo, Japan). ^1^H NMR, ^13^C NMR, DEPT, and 2D NMR spectra were recorded on an Agilent 500 MHz DD2 spectrometer (Agilent Technologies Inc., Santa Clara, CA, USA). HRESIMS spectra were obtained using a Thermo Scientific LTQ Orbitrap XL mass spectrometer (Thermo Fisher Scientific, Waltham, MA, USA). Column chromatography (CC) was achieved with silica gel (200–300 mesh, Qingdao Marine Chemical Inc., Qingdao, China) and Sephadex LH-20 (Amersham Biosciences, San Francisco, CA, USA). MPLC was done on a Bona-Agela CHEETAHTM HP100 (Beijing Agela Technologies Co., Ltd., Beijing, China). RP-HPLC was accomplished on an ODS column (HPLC (YMC-Pack ODS-A, 10 × 250 mm, 5 µm, 3 mL/min)) (YMC Co., Ltd., Kyoto, Japan).

### 3.2. Fungal Material

The fungal strain *Penicillium* sp. HDN151272 was isolated from a sample of an Antarctic associated marine sponge collected at Prydz Bay (depth 410 m, E 67.6°, S 66.1°, collected in mid-April 2016). The sponge was not identified due to the limited sample. Genetically, the fungal species was recognized by its morphological characteristics and ITS sequence of the rRNA gene. The sequence is available at GenBank with the accession number MN788660. The strain is deposited at the Key Laboratory of Marine Drugs, the Ministry of Education of China, Qingdao, People’s Republic of China.

### 3.3. Fermentation

The fungal strain *Penicillium* sp. HDN151272 was cultured and activated on PDA (potato dextrose agar) slants at 28 °C for 3 days. After activation of the strain, fermentation was achieved in Erlenmeyer flasks (500 mL) containing 150 mL of liquid culture medium, composed of glucose (1%), maltose (2%), mannitol (2%), monosodium glutamate (1%), KH_2_PO_4_ (0.05%), MgSO_4_·7H_2_O (0.03%), corn steep liquor (0.1%), and yeast extract (0.3%). The liquid culture medium was prepared by adding naturally collected seawater from Jiaozhou Bay, Qingdao, China, and adjusting the pH to 6.5. All the contents were subjected to autoclaving at 121 °C for 20 min. After autoclaving and cooling to 25 °C, each flask was inoculated with fungal spores and subjected to incubation at 28 °C for 9 days in shaking conditions.

### 3.4. Extraction and Purification

The whole fermentation broth (30 L) was filtered through a muslin cloth to separate the supernatant from the mycelia. The supernatant was extracted three times by using EtOAc (3 × 30 L), and the mycelia were homogenized and extracted three times by using MeOH (3 × 10 L). The supernatant and mycelia extracts were combined and dried in vacuo. The extract (10.0 g) was separated by VLC (vacuum liquid chromatography) on silica gel via a stepped gradient elution DCM-MeOH (100:0 to 0:100) to give twelve fractions (Fr.1 to Fr.12). Fr.5 was further separated by MPLC (C-18 ODS) using a stepped gradient elution of MeOH-H_2_O (5:95 to 100:0) to yield 11 subfractions (Fr.5-1 to Fr.5-11). Fr.5-5, Fr.5-6, and Fr.5-7 were further separated on a Sephadex LH-20 column with MeOH to provide five subfractions (Fr.5-5-1 to Fr.5-5-5), five fractions (Fr.5-6-1 to Fr.5-6-5), and four fractions (Fr.5-7-1 to Fr.5-7-4), respectively. Fr.5-5-3, Fr.5-6-4, and Fr.5-7-3 were separated by semi-preparative HPLC eluted with MeOH-H_2_O (64:36) to obtain compound **1** (7.0 mg, *t*_R_ = 33.3 min), MeOH-H_2_O (43:57) to obtain compound **2** (6.0 mg, *t*_R_ = 38.2 min), MeOH-H_2_O (40:60) to obtain **3** (5.5 mg, *t*_R_ = 31.8 min), respectively.

**Ketidocillinone A (1)**. deep yellowish powder; UV (MeCN) *λ*max (log *ε*): 241 (3.16) nm; IR (KBr) *ν*_max_ 3276, 2929, 1698, 1653, 1203, 1139, 1026, 801, 723 cm^−1^; ^1^H and ^13^C NMR data see Table 1 and Table 2; HRESIMS *m*/*z* 247.0981 [M − H]^−^ (calcd. for C_14_H_15_O_4_, 247.0976), *m*/*z* 249.1134 [M − H]^−^ (calcd. for C_14_H_17_O_4_, 247.1132).

**Ketidocillinone B (2)**. deep yellowish powder; UV (MeCN) *λ*max (log *ε*): 241 (3.32) nm; IR (KBr) *ν*_max_ 3355, 2922, 2378, 1698, 1498, 1208, 1136, 1030, 840, 802, 723 cm^−1^; ^1^H and ^13^C NMR data see Table 1 and Table 2; HRESIMS *m*/*z* 261.1129 [M − H]^−^ (calcd. for C_15_H_17_O_4_, 261.1132), *m*/*z* 263.1289 [M − H]^−^ (calcd. for C_15_H_19_O_4_, 263.1289).

**Ketidocillinone C (3)**. light yellowish powder; ${\left[\alpha \right]}_{D}^{20}$ +4.56 (*c* 0.03, MeOH); UV (MeCN) *λ*max (log *ε*): 242 (2.56) nm; IR (KBr) *ν*_max_ 3338, 2929, 2358, 1717, 1698, 1457, 1205, 1142, 1027, 874, 801, 722, 518 cm^−1^; ECD (2.5 mM, MeOH) *λ*max (*Δε*) 210 (+4.24), 242 (−4.56), 342 (+5.25) nm; ^1^H and ^13^C NMR data see Table 1 and Table 2; HRESIMS *m*/*z* 235.0975 [M − H]^−^ (calcd. for C_13_H_15_O_4_, 235.0976), *m*/*z* 237.1131 [M − H]^−^ (calcd. for C_13_H_17_O_4_, 237.1132).

### 3.5. Assay of Antimicrobial Activity

Antimicrobial activity was determined by the broth microdilution method [13] against eight types of bacterial strains such as MRCNS, *Vibrio parahemolyticus*, *Escherichia coli*, *Proteus species*, *Bacillus subtilis*, *Bacillus cereus*, *Mycobacterium phlei*, *Pseudomonas aeruginosa*, and one fungus *Monilia albican* and were used in the antimicrobial assay. All experiments were performed in triplicates, and ciprofloxacin (J&K Chemical Technology, Beijing, China) was used as a positive control. All strains were donated by Qingdao municipal hospital and deposited at the Key Laboratory of Marine Drugs, the Ministry of Education of China, Qingdao, People’s Republic of China.

### 3.6. Computation Section

Conformational searches were run employing the “systematic” procedure implemented in Spartan 14 [14], using MMFF (merck molecular force field). All MMFF minima were reoptimized with DFT calculations at the B3LYP/6-31+G(d) level using the Gaussian09 program [15]. The geometry was optimized, starting from various initial conformations, with vibrational frequency calculations confirming the presence of minima. TDDFT (time-dependent DFT) calculations were performed on the six lowest-energy conformations for **3** (>5% population) for each configuration using 20 excited states and using a PCM (polarizable continuum model) for MeOH. ECD spectra were generated using the program SpecDis [16] by applying a Gaussian band shape with 0.15 eV from dipole-length rotational strengths. The dipole velocity forms yielded negligible differences. The spectra of the conformers were combined using Boltzmann weighting, with the lowest-energy conformations accounting for about 99% of the weights. The calculated spectrum was blue-shifted by 10 nm to facilitate comparison to the experimental data.

## 4. Conclusions

The prenylated quinones and hydroquinones are omnipresent in natural surroundings [17] and extensively isolated from marine sponges [18,19], marine algae [20,21,22], ascidians [23,24,25], and microbes [26,27]. They have been spotted to have a diverse range of bioactivities, including cytotoxic [27,28], antioxidant [21,28], and anti-inflammatory [25] activities, among which quinone and hydroquinone nucleus seemed to be the critical pharmacophore [28]. Three new hydroquinone derivatives, ketidocillinones A–C (**1**−**3**), were obtained from the Antarctica-sponge derived fungus *Penicillium* sp. HDN151272. Compounds **2** and **3** exhibited broad-spectrum antibacterial activities, especially against MRCNS and *Mycobacterium phlei*, which provide potential candidates for antibacterial drug development.

## Figures and Tables

**Table 1:** ^1^H (500 MHz, *J* in Hz) NMR and ^13^C (125 MHz) NMR data for compounds **1**−**3** in DMSO-*d*_6_.

No.	1		2		3	
	^1^H	^13^C (Type)	^1^H	^13^C (Type)	^1^H	^13^C (Type)
1		147.5, C		147.5, C		147.5, C
2		126.0, C		125.9, C		125.4, C
3	6.44, s	116.2, CH	6.44, s	117.4, CH	6.41, s	116.2, CH
4		147.9, C		147.9, C		147.9, C
5		121.7, C		121.8, C		121.9, C
6	6.44, s	117.5, CH	6.43, s	116.3, CH	6.44, s	117.5, CH
7	2.48, m	28.7, CH_2_	2.46, m	28.6, CH_2_	2.36, ov.	27.3, CH_2_
8	2.12, m	39.9, CH_2_	2.13, t (8.5)	39.8, CH_2_	1.53, m	33.9, CH_2_
					1.75, m	
9		138.2, C		138.9, C	2.36, ov.	38.6, CH
10	5.26, t (7.2)	116.8, CH	5.24, m	116.1, CH		176.7, C
11	2.93, d (7.1)	33.7, CH_2_	3.03, d (7.1)	33.3, CH_2_	3.58, s	51.7, CH_3_
12		173.5, C		172.4, C	1.97, s	16.2, CH_3_
13			3.57, s	51.8, CH_3_	1.08, s	17.2, CH_3_
14	1.60, s	16.7, CH_3_	1.61, s	16.7, CH_3_		
15	1.97, s ov.	16.2,CH_3_	1.97, s	16.2, CH_3_		
1-OH	8.34, s		8.35, s		8.35, s	
4-OH	8.30, s		8.31, s		8.33, s	

**Table 2:** ^1^H (500 MHz, *J* in Hz) NMR data for compounds **1**–**3** in CDCl_3_.

No.	1		2		3	
	a	b	a	b	a	b
3	6.53, ov.	6.53, ov.	6.55, ov.	6.55, ov.	6.55, s	6.52, s
6	6.53, ov.	6.53, ov.	6.55, ov.	6.55, ov.	6.59, s	6.62, s
7	2.51, t (7.5)	2.62, m	2.53, t (7.4)	2.65, t (7.5)	2.42, m	2.52, ov.
8	2.20, t (7.5)	2.26, t (7.8)	2.23, t (7.4)	2.28, t (7.5)	1.88, ov.	1.89, ov.
					1.62, m	1.71, m
9					2.51, ov.	2.51, ov.
10	5.32, ov.	5.28, ov.	5.32, t (6.4)	5.28,t (6.9)		
11	3.02, ov.	3.02, ov.	3.04, ov.	3.04, ov.	3.68, s	3.72, s
12					2.04, s	2.17, s
13			3.67, s	3.68, s	1.19, ov.	1.20, ov.
14	2.00, s	2.12, s	2.03, s	2.16, s		
15	1.64, s	1.65, s	1.66, s	1.68, s		

ov. Overlapped signals.

**Table 3:** ^13^C (125 MHz) NMR data for compounds **1**–**3** in CDCl_3_.

No.	1 (Type)		2 (Type)		3 (Type)	
	a	b	a	b	a	b
1	147.6, C	185.9, C	146.9, C	187.6, C	147.7, C	187.5, C
2	127.0, C	148.8, C	126.7, C	148.7, C	125.6, C	148.6, C
3	117.6, CH	132.9, CH	116.7, CH	132.8, CH	116.3, CH	132.7, CH
4	147.7, C	187.7, C	147.6, C	188.1, C	147.3, C	188.1, C
5	122.1, C	145.6, C	122.2, C	145.6, C	122.7, C	145.7, C
6	118.0, CH	133.6, CH	118.0, CH	133.5, CH	118.3, CH	133.6, CH
7	29.8, CH_2_	27.2, CH_2_	27.9, CH_2_	27.1, CH_2_	27.6, CH_2_	26.5, CH_2_
8	39.8, CH_2_	37.7, CH_2_	39.7, CH_2_	37.6, CH_2_	34.0, CH_2_	31.5, CH_2_
9	138.2, C	137.3, C	139.2, C	137.3, C	38.7, CH	39.0, CH
10	116.9, CH	118.0, CH	116.3, CH	117.2, CH	177.9, C	176.5, C
11	33.6, CH_2_	33.4, CH_2_	33.5, CH_2_	33.5, CH_2_	52.0, CH_3_	51.7, CH_3_
12	173.7, C	171.9, C	173.2, C	172.5, C	15.5, CH_3_	15.5, CH_3_
13			51.9, CH_3_	51.8, CH_3_	17.3, CH_3_	17.1, CH_3_
14	15.5, CH_3_	15.7, CH_3_	15.4, CH_3_	15.5, CH_3_		
15	16.7, CH_3_	16.3, CH_3_	16.5, CH_3_	16.2, CH_3_		

**Table 4:** Antibacterial activity for compounds **1**–**3** (mixtures of the phenol and quinone products) (MIC, µg/mL).

Compd.	*V. Parahemolyticus*	*E. coli*	*Prot* *-* *eus sp.*	*B. subtilis*	*MRCNS*	*B. cereus*	*P. aeruginosa*	*M. Phlei*	*M. albican*
**1**	>50	>50	>50	>50	>50	>50	>50	>50	>50
**2**	>50	>50	>50	>50	6.25	12.50	1.56	3.13	>50
**3**	12.50	>50	>50	12.50	6.25	25.00	6.25	6.25	>50
Ciprofloxacin	0.52	2.07	0.52	0.98	0.98	0.98	0.98	3.91	3.91

## Supplementary Materials

The following are available online at https://www.mdpi.com/1660-3397/18/2/71/s1. Figure S1: HPLC analysis of the crude of HDN151272; Figure S2: The 18S rDNA sequences data of *Penicillium* sp. HDN151272; Figure S3–S12: 1D and 2D NMR spectra of compound **1** in DMSO-*d*_6_; HRESIMS spectrum, IR spectrum, UV spectrum of compound **1**; Figure S13–S21: 1D and 2D NMR data in DMSO-*d*_6_, HRESIMS spectrum, IR spectrum, UV spectrum of compound **2**; Figure S23–S31: 1D and 2D NMR data of compound **3** in DMSO-*d*_6_. HRESIMS spectrum, IR spectrum, UV spectrum of compound **3**; Figure S32–S38: 1D and 2D NMR spectra of compound **1** in CDCl_3_; Figure S39–S45: 1D and 2D NMR spectra of compound **2** in CDCl_3_; Figure S46–S51: 1D and 2D NMR spectra of compound **3** in CDCl_3_.
