# Supplementary material for: Antibacterial Polyketides from Antarctica Sponge-Derived Fungus Penicillium sp. HDN151272
Source: Mar Drugs. 2020 Jan 23;18(2):71. doi: 10.3390/md18020071 (PMC7073682; doi:10.3390/md18020071)

Supporting Information for

Antibacterial Polyketides from Antarctica Sponge-derived Fungus *Penicillium* sp. HDN151272

Mudassir Shah [1,a], Chunxiao Sun [1,a], Zichao sun [1], Guojian Zhang [1,2], Qian Che [1], Tianjiao Zhu [1*], Qianqun Gu [1], Dehai Li [1,2,3*]

[1] Key Laboratory of Marine Drugs, Chinese Ministry of Education, School of Medicine and Pharmacy, Ocean University of China, Qingdao 266003, P. R. China.
[2] Laboratory for Marine Drugs and Bioproducts, Pilot National Laboratory for Marine Science and Technology, Qingdao, 266237, P. R. China
[3] Open Studio for Druggability Research of Marine Natural Products, Pilot National Laboratory for Marine Science and Technology, Qingdao, 266237, P. R. China; dehaili@ouc.edu.cn
[*] Correspondence: dehaili@ouc.edu.cn (D.L.), zhutj@ouc.edu.cn (T. Z.); Tel.: 0086-532-82031619
[a] Contributed equally.

Table of Contents

Figure S1. HPLC analysis of the crude of HDN151272. 4

Figure S2. The 18S rDNA sequences data of *Penicillium* sp. HDN151272. 4

Figure S3. ^{1}H NMR (500 MHz, DMSO-d_6) spectrum of compound **1**. 5

Figure S4. ^{13}C NMR (125 MHz, DMSO-d_6) spectrum of compound **1**. 5

Figure S6. ^{1}H-^{1}H COSY spectrum of compound **1** in DMSO-d_6. 6

Figure S7. HSQC spectrum of compound **1** in DMSO-d_6. 7

Figure S8. ^{1}H-^{13}C HMBC spectrum of compound **1** in DMSO-d_6. 7

Figure S9. NOESY spectrum of compound **1** in DMSO-d_6 8

Figure S10. HRESIMS spectrum of compound **1**. 8

Figure S11. IR spectrum of compound **1**. 9

Figure S12. UV spectrum of compound **1**. 9

Figure S13. ^{1}H NMR (500 MHz, DMSO-d_6) spectrum of compound **2**. 10

Figure S14. ^{13}C NMR (125 MHz, DMSO-d_6) spectrum of compound **2**. 10

Figure S15. DEPT (125 MHz, DMSO-d_6) spectrum of compound **2**. 11

Figure S16. ^{1}H-^{1}H COSY spectrum of compound **2** in DMSO-d_6. 11

Figure S17. HSQC spectrum of compound **2** in DMSO-d_6. 12

Figure S18. ^{1}H-^{13}C HMBC spectrum of compound **2** in DMSO-d_6. 12

Figure S19. HRESIMS spectrum of compound **2**. 13

Figure S20. IR spectrum of compound **2**. 13

Figure S21. UV spectrum of compound **2**. 14

Figure S23. ^{13}C NMR (125 MHz, DMSO-d_6) spectrum of compound **3**. 15

Figure S24. DEPT (125 MHz, DMSO-d_6) spectrum of compound **3**. 15

Figure S25. ^{1}H-^{1}H COSY spectrum of compound **3** in DMSO-d_6. 16

Figure S26. HSQC spectrum of compound **3** in DMSO-d_6. 16

Figure S27. ^{1}H-^{13}C HMBC spectrum of compound **3** in DMSO-d_6. 17

Figure S28. NOESY spectrum of compound **3** in DMSO-d_6. 17

Figure S29. HRESIMS spectrum of compound **3**. 18

Figure S30. IR spectrum of compound **3**. 18

Figure S31. UV spectrum of compound **3**. 19
Figure S32. ^{1}H NMR (500 MHz, $CDCl_3$) spectrum of compound **1**. 19
Figure S33. ^{13}C NMR (125 MHz, $CDCl_3$) spectrum of compound **1**. 20
Figure S34. DEPT (125 MHz, $CDCl_3$) spectrum of compound **1**. 20
Figure S35. ^{1}H-^{1}H COSY spectrum of compound **1** in $CDCl_3$. 21
Figure S36. ^{1}H-^{13}C HSQC spectrum of compound **1** in $CDCl_3$. 21
Figure S37. ^{1}H-^{13}C HMBC spectrum of compound **1** in $CDCl_3$. 22
Figure S38. NOESY spectrum of compound **1** in $CDCl_3$. 22
Figure S39. ^{1}H NMR (500 MHz, $CDCl_3$) spectrum of compound **2**. 23
Figure S40. ^{13}C NMR (125 MHz, $CDCl_3$) spectrum of compound **2**. 23
Figure S41. DEPT (125 MHz, $CDCl_3$) spectrum of compound **2**. 24
Figure S42. ^{1}H-^{1}H COSY spectrum of compound **2** in $CDCl_3$. 24
Figure S43. HSQC spectrum of compound **2** in $CDCl_3$. 25
Figure S44. ^{1}H-^{13}C HMBC spectrum of compound **2** in $CDCl_3$. 25
Figure S45. NOESY spectrum of compound **2** in $CDCl_3$. 26
Figure S46. ^{1}H NMR (500 MHz, $CDCl_3$) spectrum of compound **3**. 26
Figure S47. ^{13}C NMR (125 MHz, $CDCl_3$) spectrum of compound **3**. 27
Figure S48. DEPT (125 MHz, $CDCl_3$) spectrum of compound **3**. 27
Figure S49. ^{1}H-^{1}H COSY spectrum of compound **3** in $CDCl_3$. 28
Figure S50. HSQC spectrum of compound **3** in $CDCl_3$. 28
Figure S51. ^{1}H-^{13}C HMBC spectrum of compound **3** in $CDCl_3$. 29

Figure S1. HPLC analysis of the crude of HDN151272.

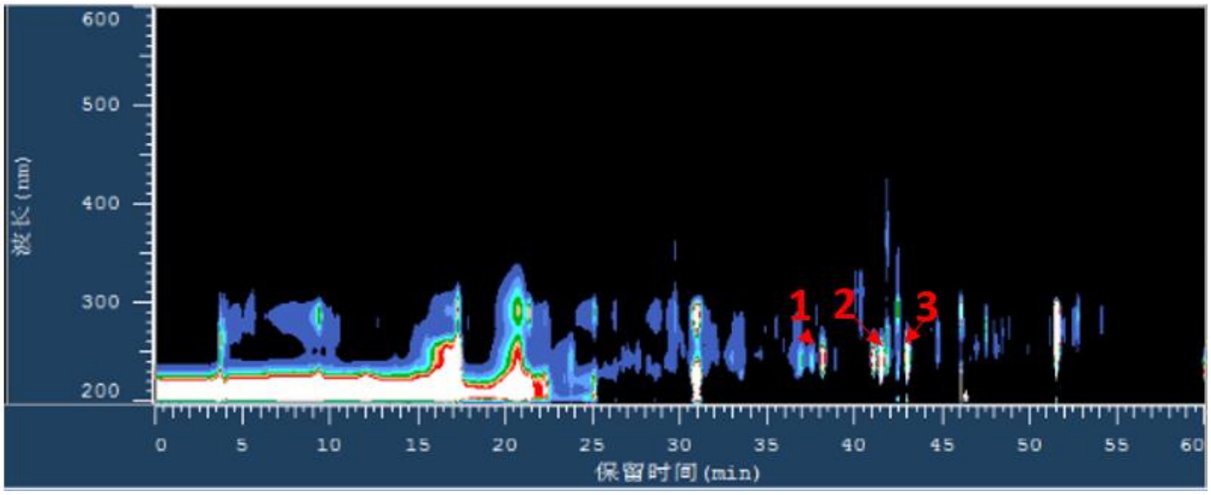

Figure S2. The 18S rDNA sequences data of *Penicillium* sp. HDN151272.

TGGCTCGACGAGGCTCTGGGTCACCTCCCACCCGTGTTTATTTTACCTTGTTGCTTCGGCGGGCCC
GCCTTAACTGGCCGCCGGGGGGCTTACGCCCCCGGGCCCGCGCCCGCCGAAGACACCCTCGAAC
TCTGTCTGAAGATTGTAGTCTGAGTGAAAATATAAATTATTTAAAACTTTCAACAACGGATCTCTT
GGTTCCGGCATCGATGAAGAACGCAGCGAAATGCGATACGTAATGTGAATTGCAAATTCAGTGA
ATCATCGAGTCTTTGAACGCACATTGCGCCCCCTGGTATTCCGGGGGGCATGCCTGTCCGAGCGT
CATTTCTGCCCTCAAGCACGGCTTGTGTGTTGGGCCCCGTCCTCCGATCCCGGGGGACGGGCCCG
AAAGGCAGCGGCGGCACCGCGTCCGGTCCTCGAGCGTATGGGGCTTTGTCACCCGCTCTGTAGGC
CCGGCCGGCGCTTGCCGATCAACCCAAATTTTTATCCAGGTTGACCTCGGATCAGGTAGGGATAC
CCGCTGAACTTAAGCATATCAATAAGCGGAGGAA

Figure S3. ^{1}H NMR (500 MHz, DMSO-d_6) spectrum of compound **1**.

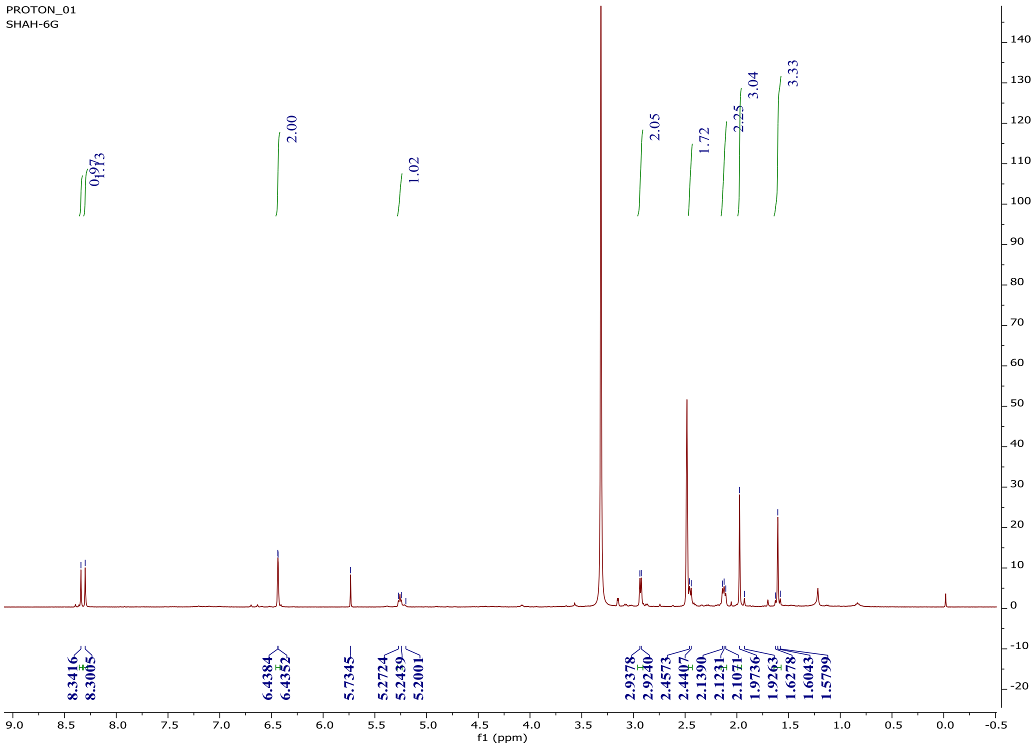

Figure S4. ^{13}C NMR (125 MHz, DMSO-d_6) spectrum of compound **1**.

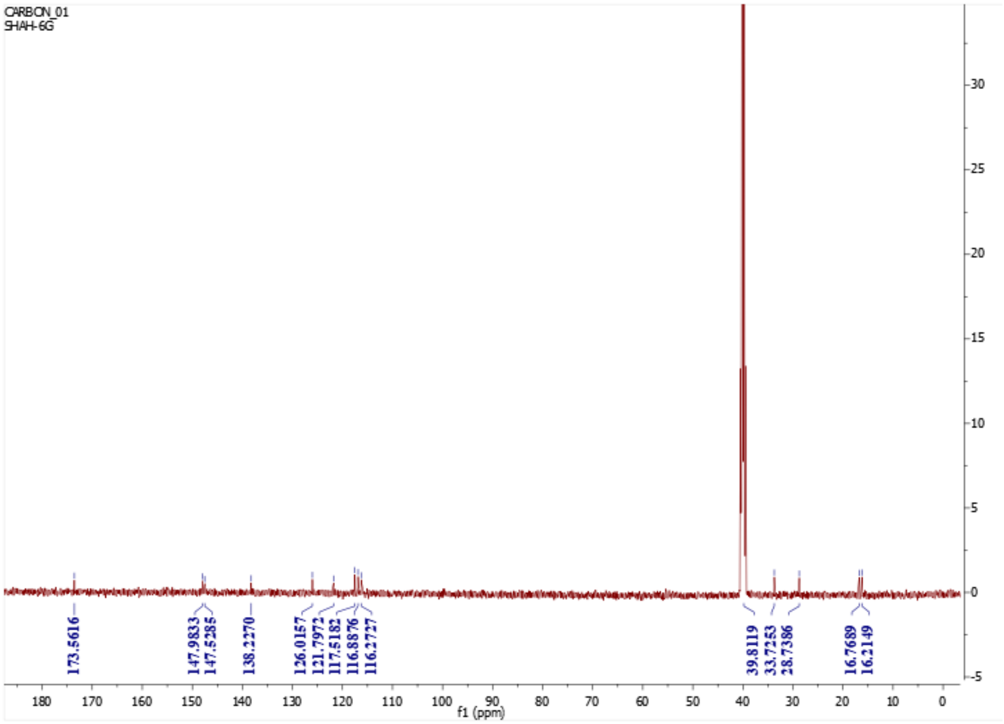

Figure S5. DEPT (125 MHz, DMSO-d_6) spectrum of compound **1**

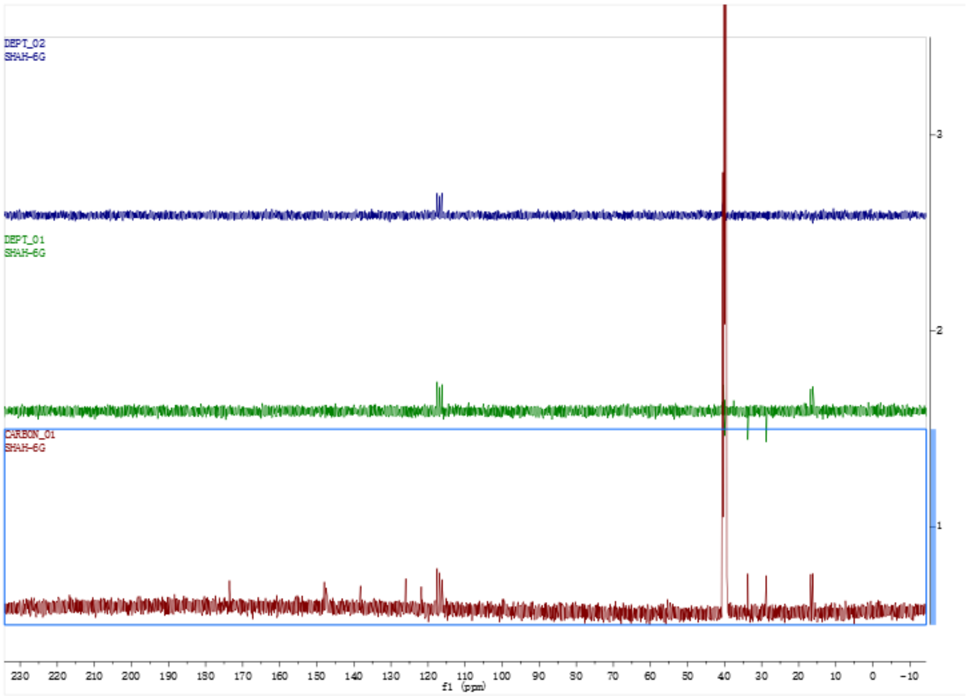

Figure S6. ^{1}H-^{1}H COSY spectrum of compound **1** in DMSO-d_6.

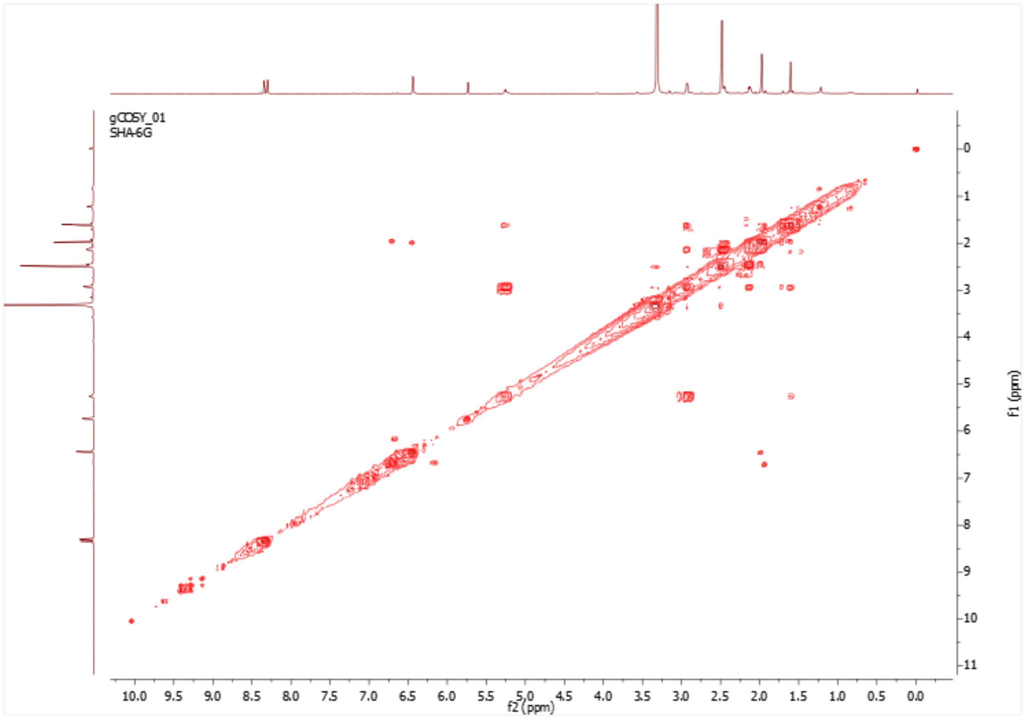

Figure S7. HSQC spectrum of compound **1** in DMSO-d_6.

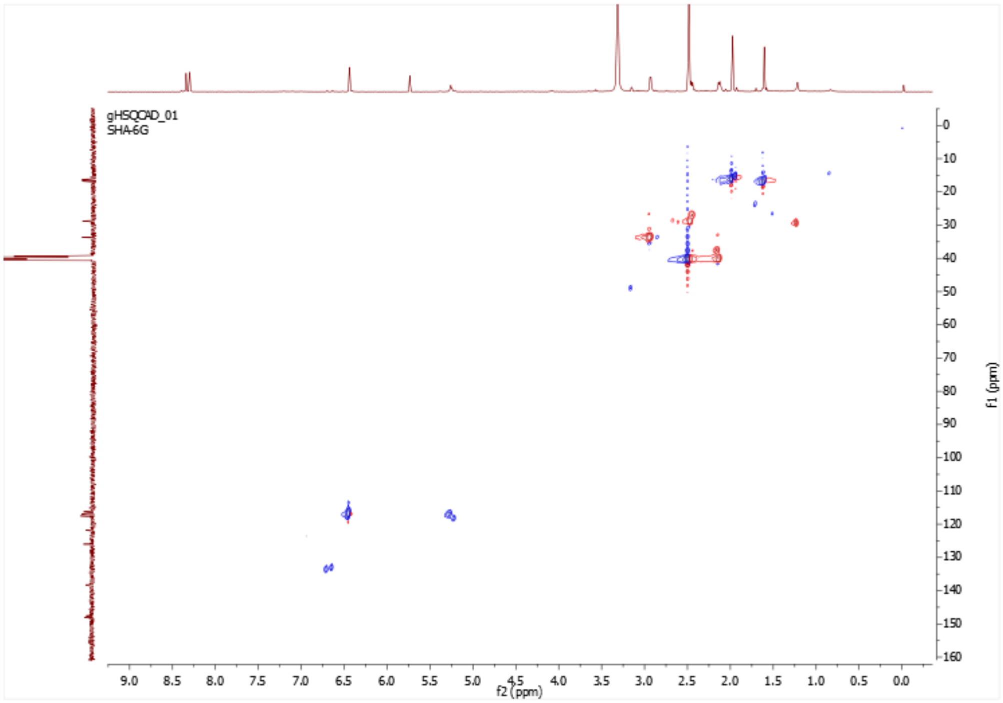

Figure S8. ^{1}H-^{13}C HMBC spectrum of compound **1** in DMSO-d_6

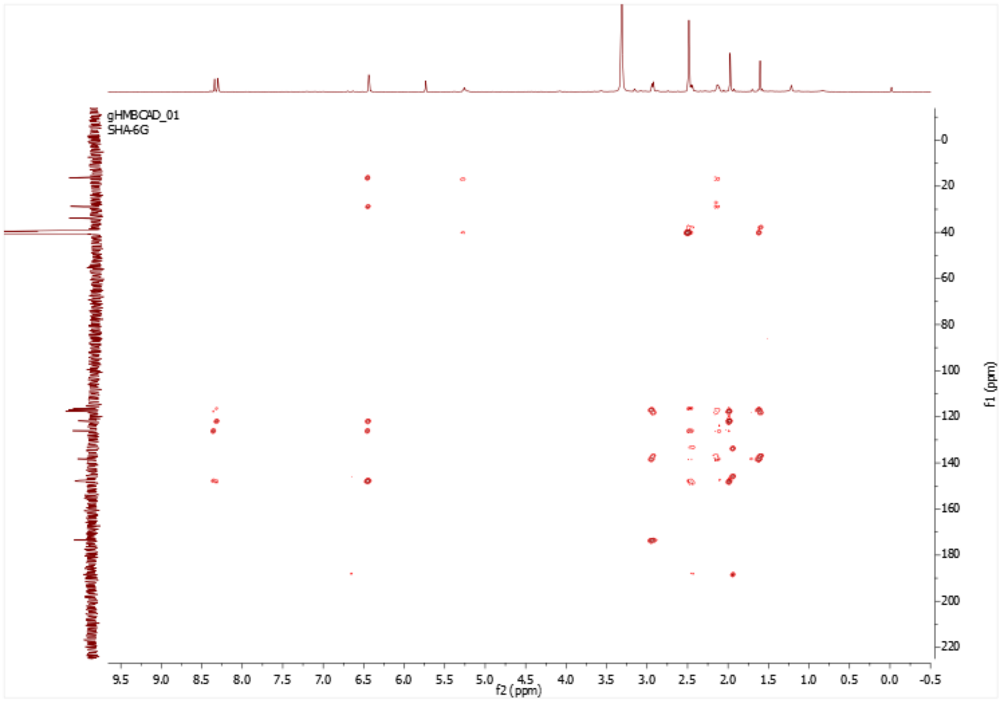

Figure S9. NOESY spectrum of compound **1** in DMSO-d_6

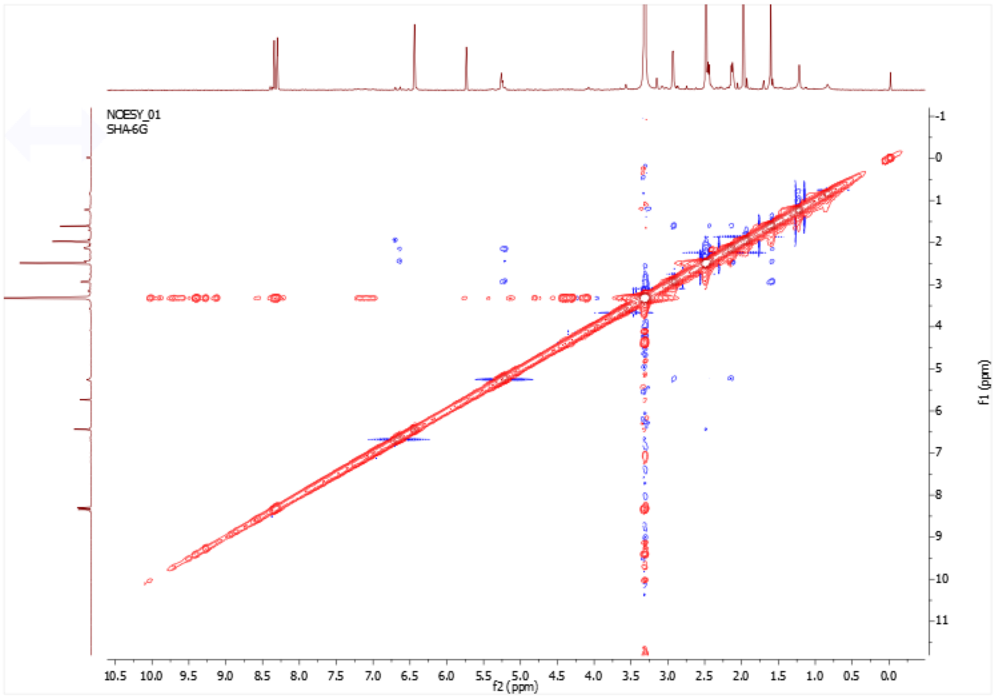

Figure S10. HRESIMS spectrum of compound **1**.

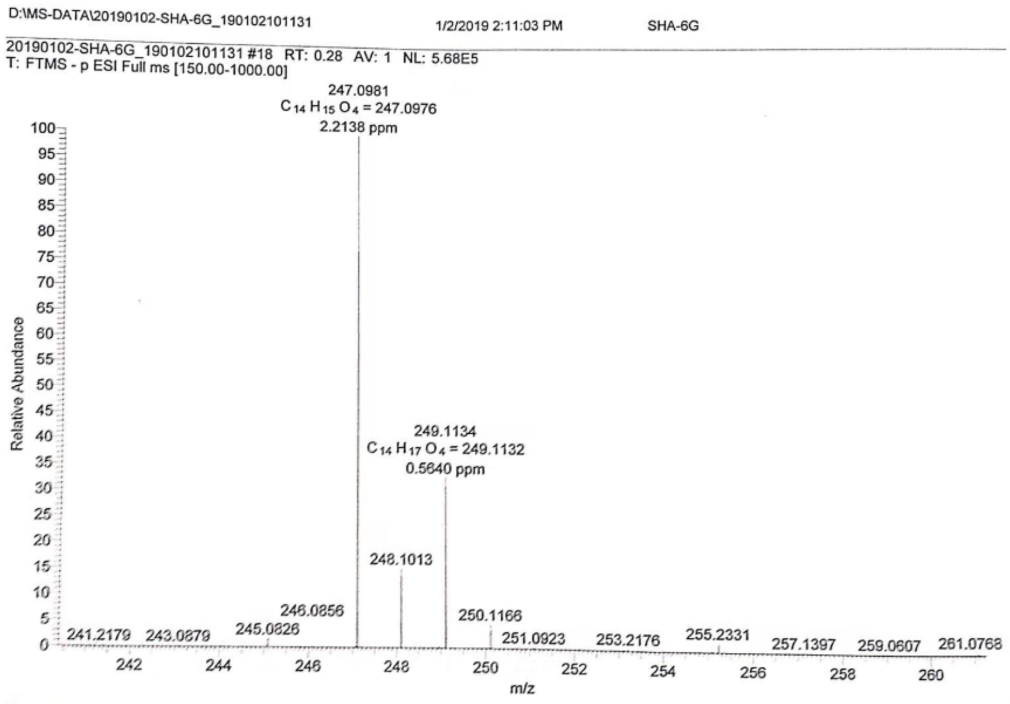

Figure S11. IR spectrum of compound **1**.

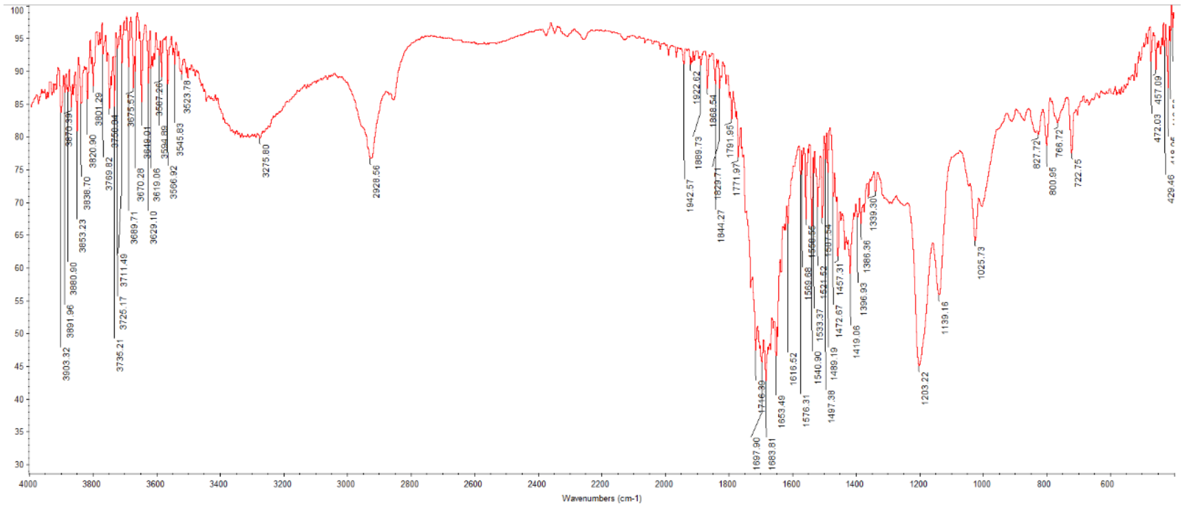

Figure S12. UV spectrum of compound **1**.

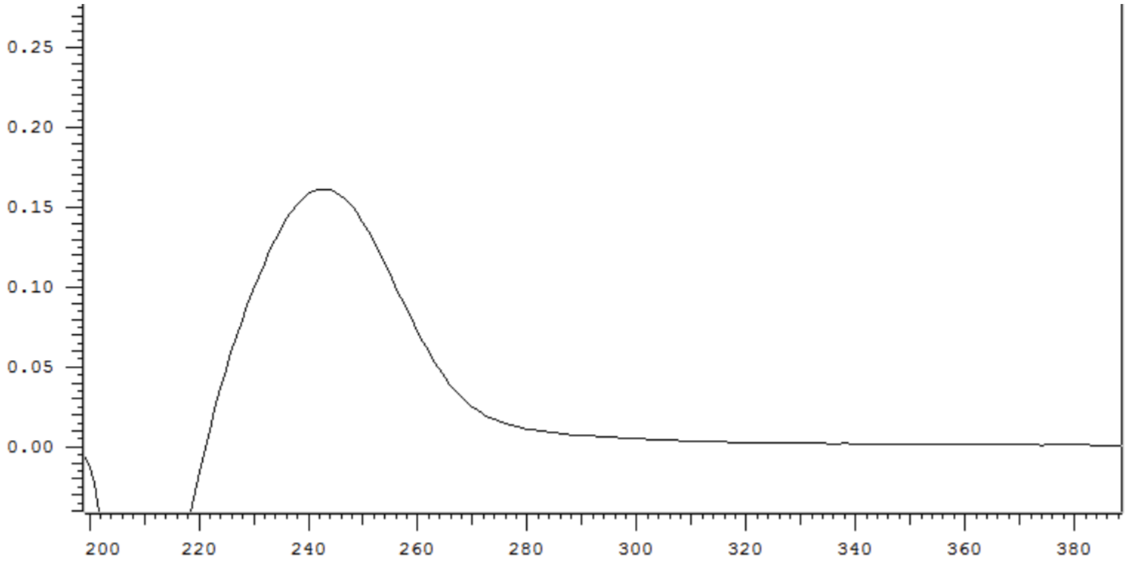

Figure S13. ^{1}H NMR (500 MHz, DMSO-d_6) spectrum of compound **2**.

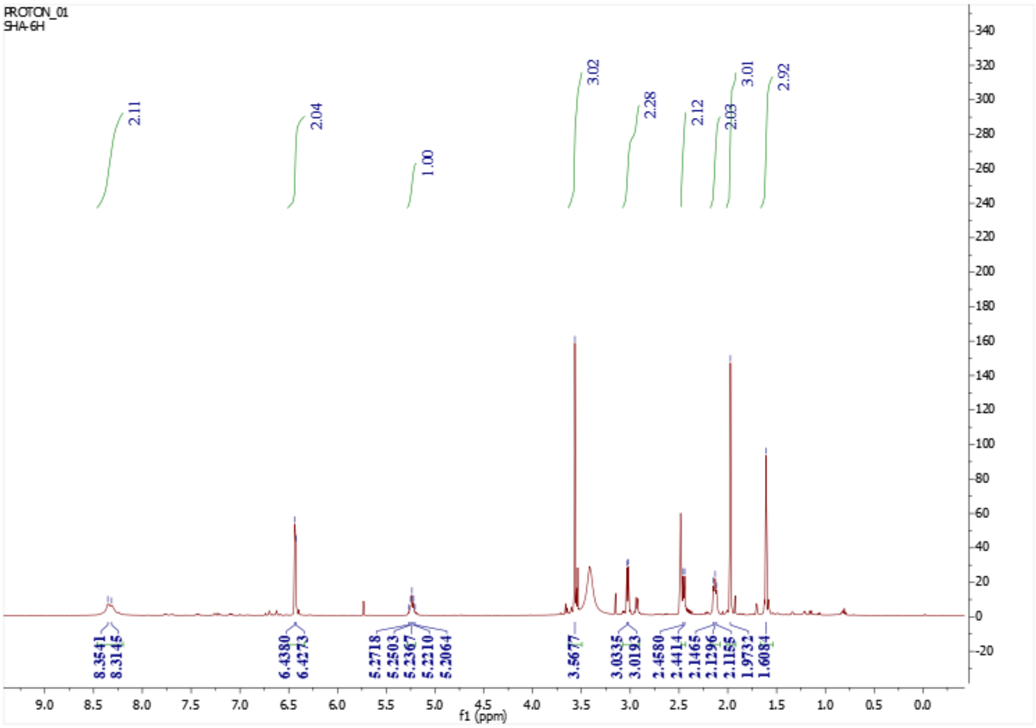

Figure S14. ^{13}C NMR (125 MHz, DMSO-d_6) spectrum of compound **2**.

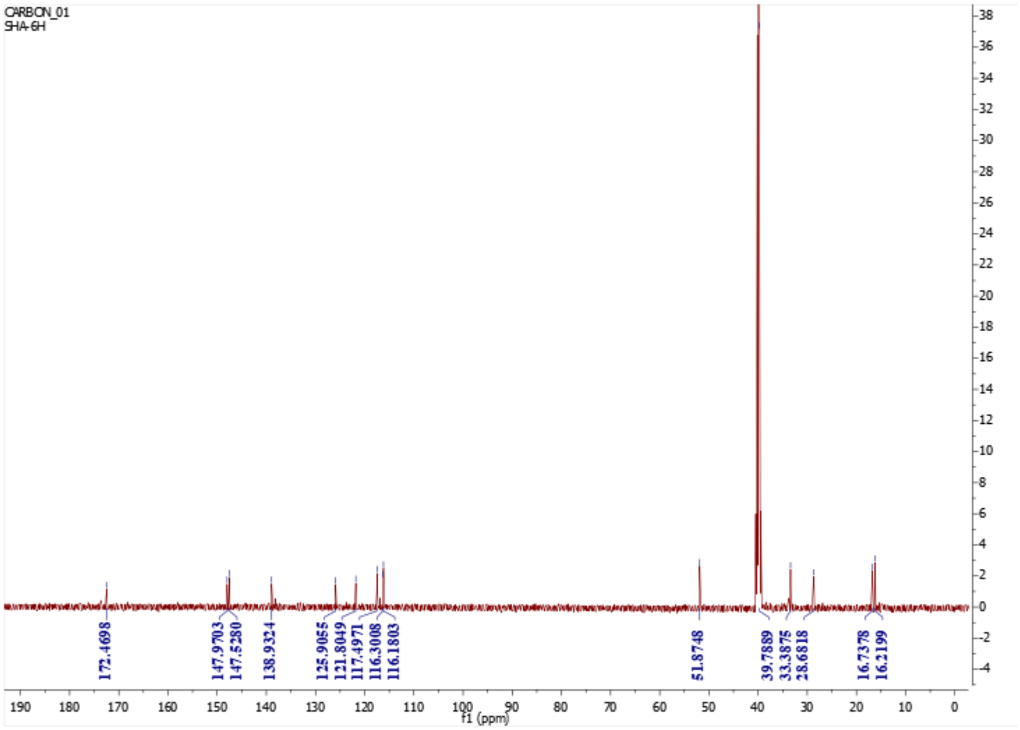

Figure S15. DEPT (125 MHz, DMSO-d_6) spectrum of compound **2**.

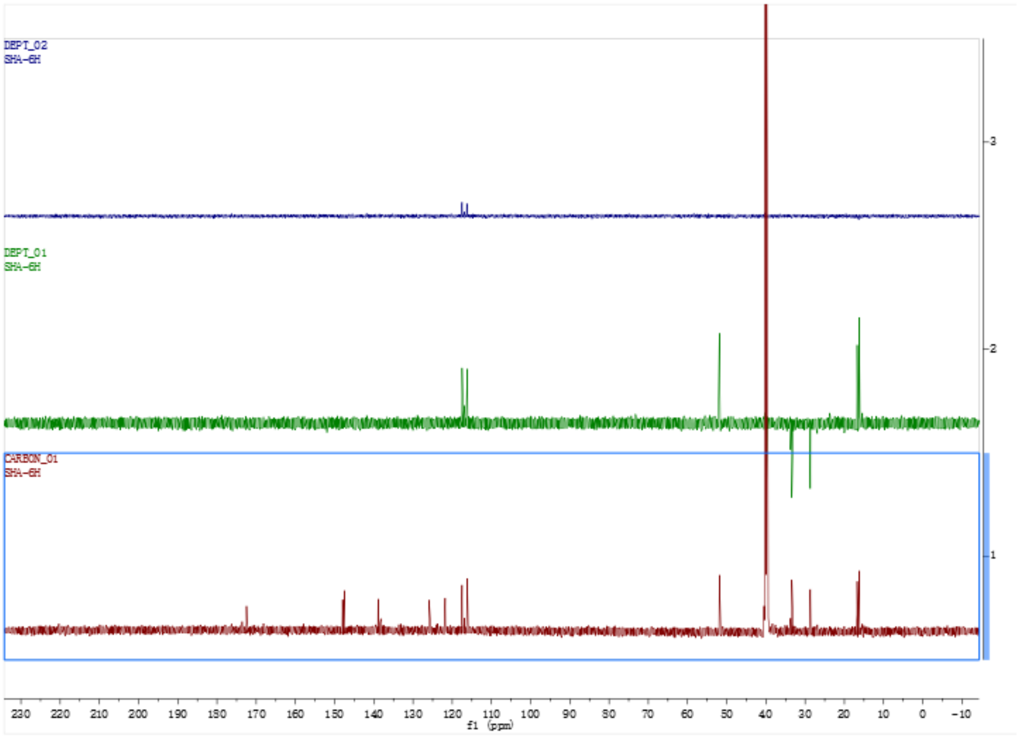

Figure S16. ^{1}H-^{1}H COSY spectrum of compound **2** in DMSO-d_6.

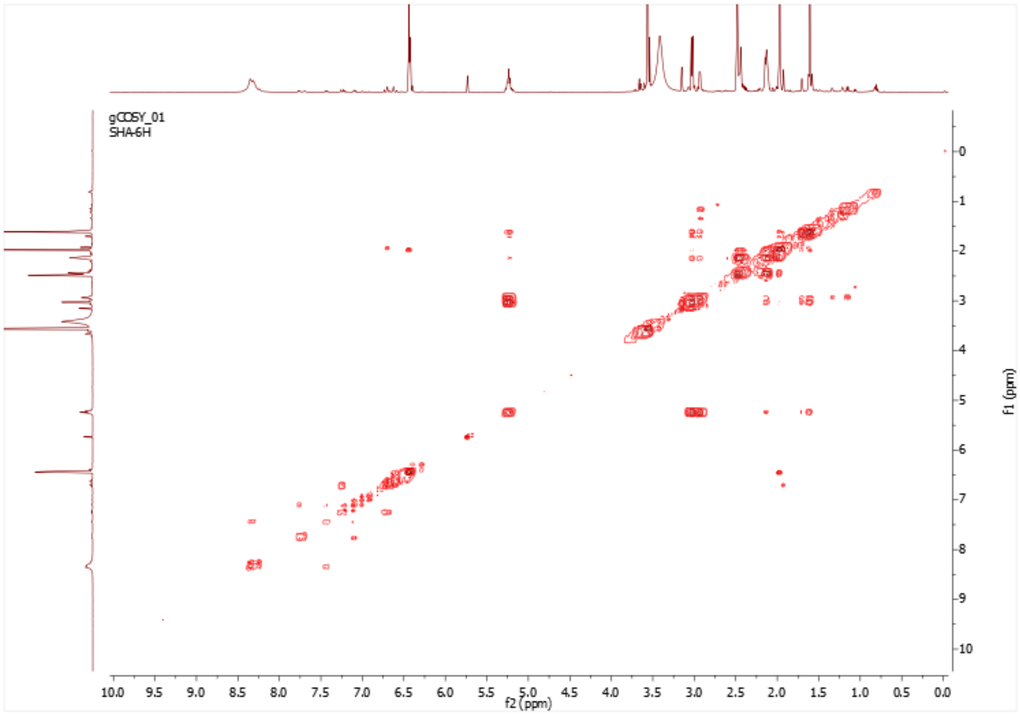

Figure S17. HSQC spectrum of compound **2** in DMSO-d_6.

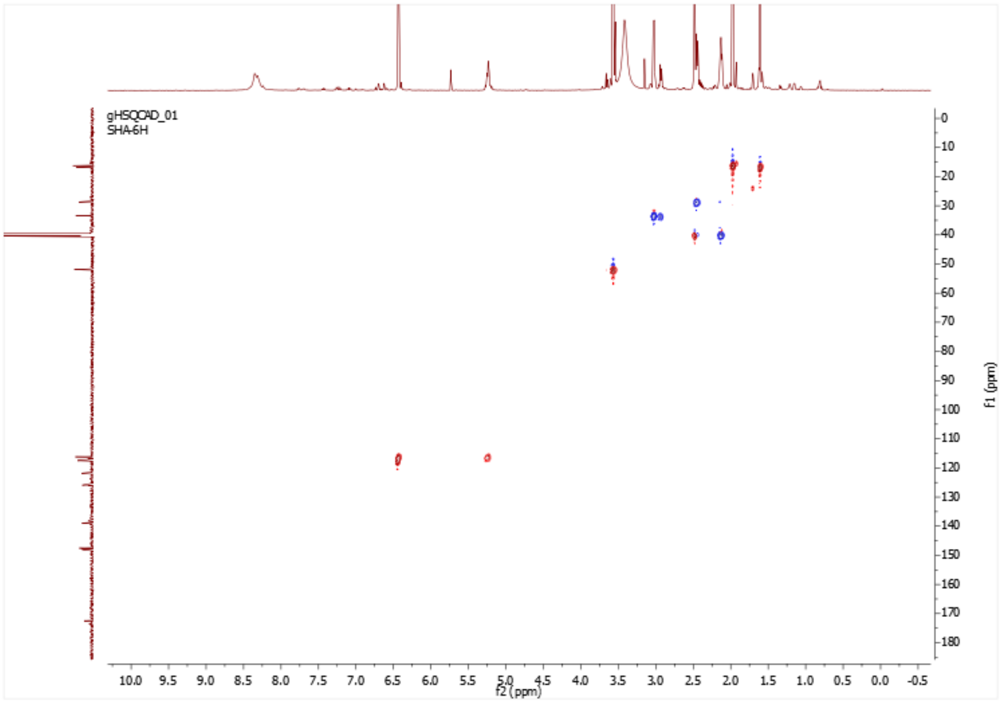

Figure S18. ^{1}H-^{13}C HMBC spectrum of compound **2** in DMSO-d_6.

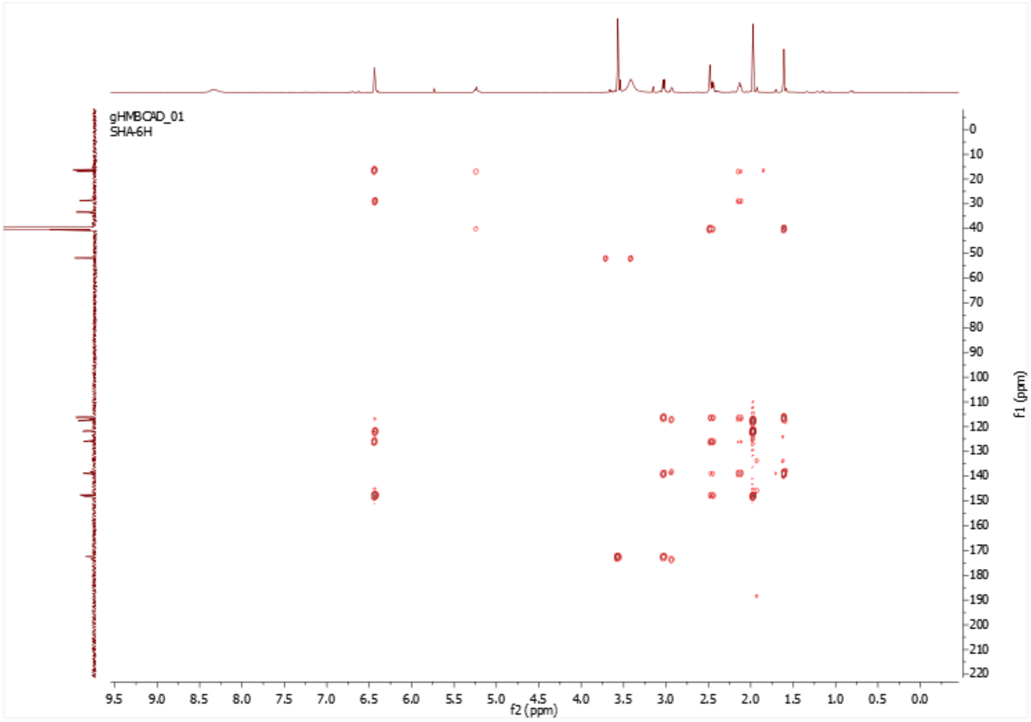

Figure S19. HRESIMS spectrum of compound **2**.

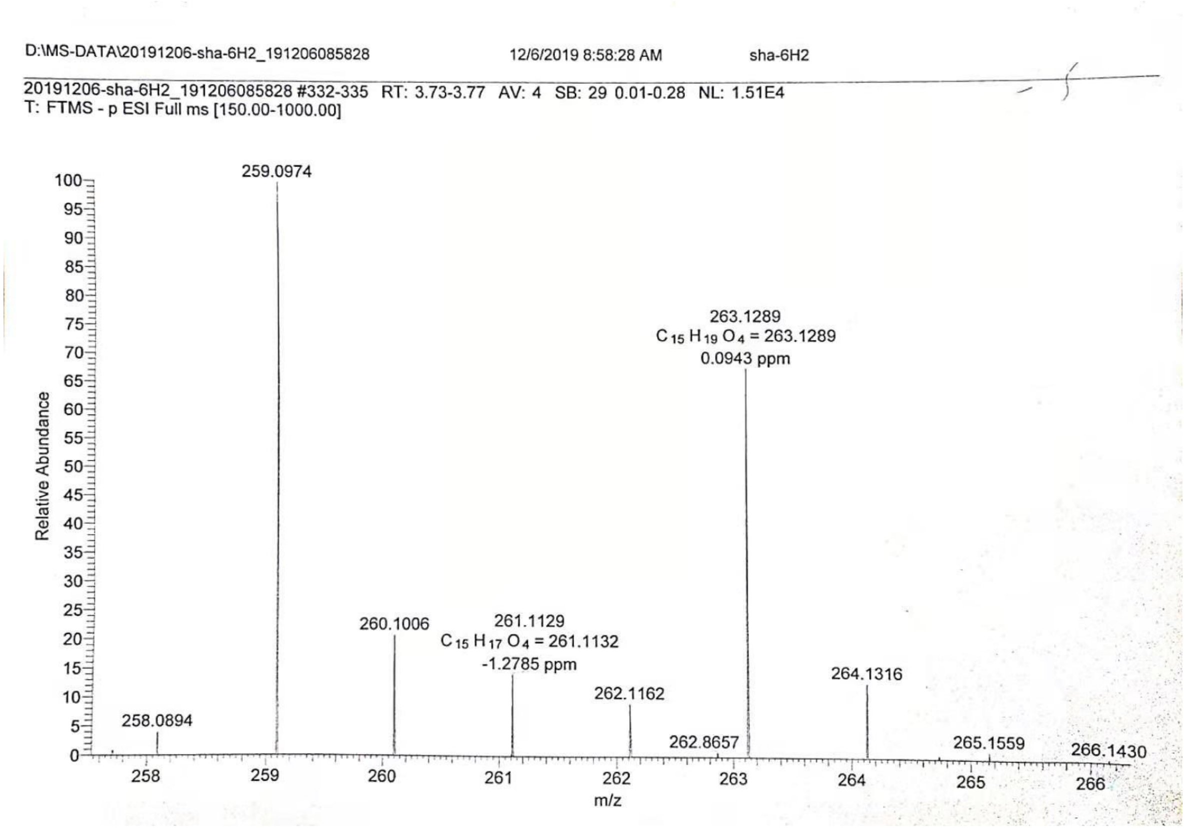

Figure S20. IR spectrum of compound **2**.

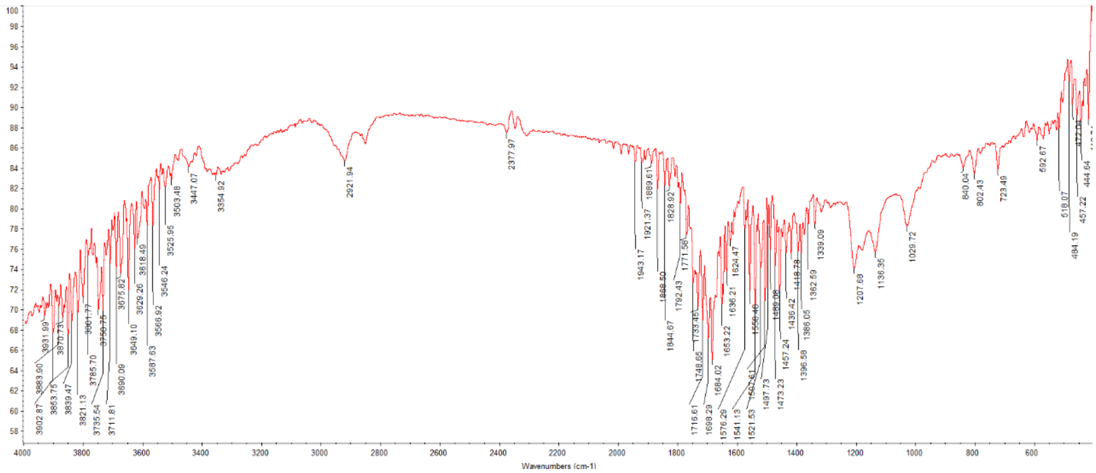

Figure S21. UV spectrum of compound **2**.

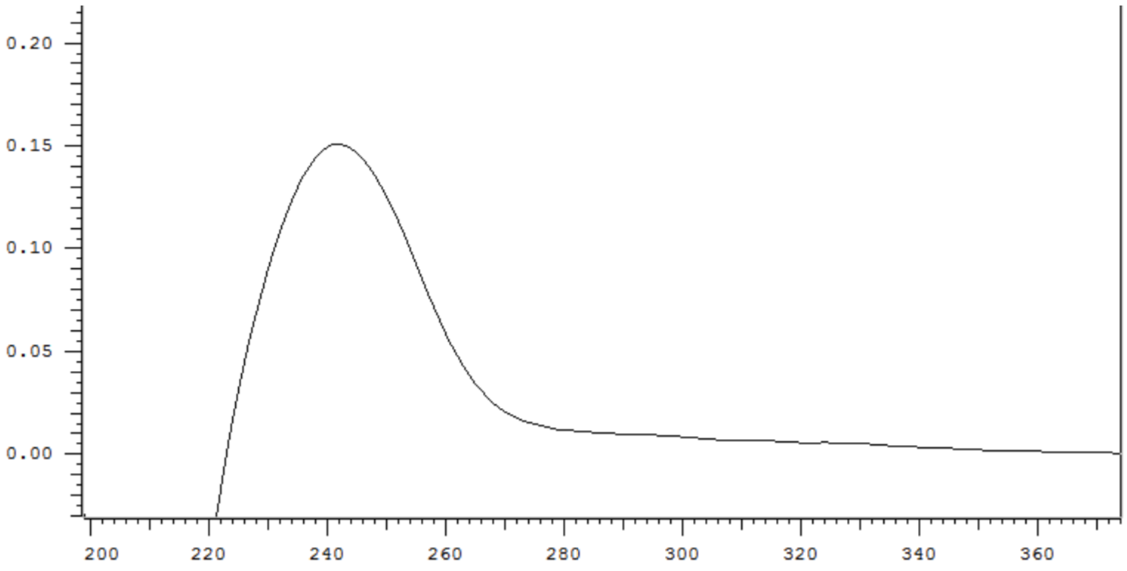

Figure S22. ^{1}H NMR (500 MHz, DMSO-d_6) spectrum of compound **3**.

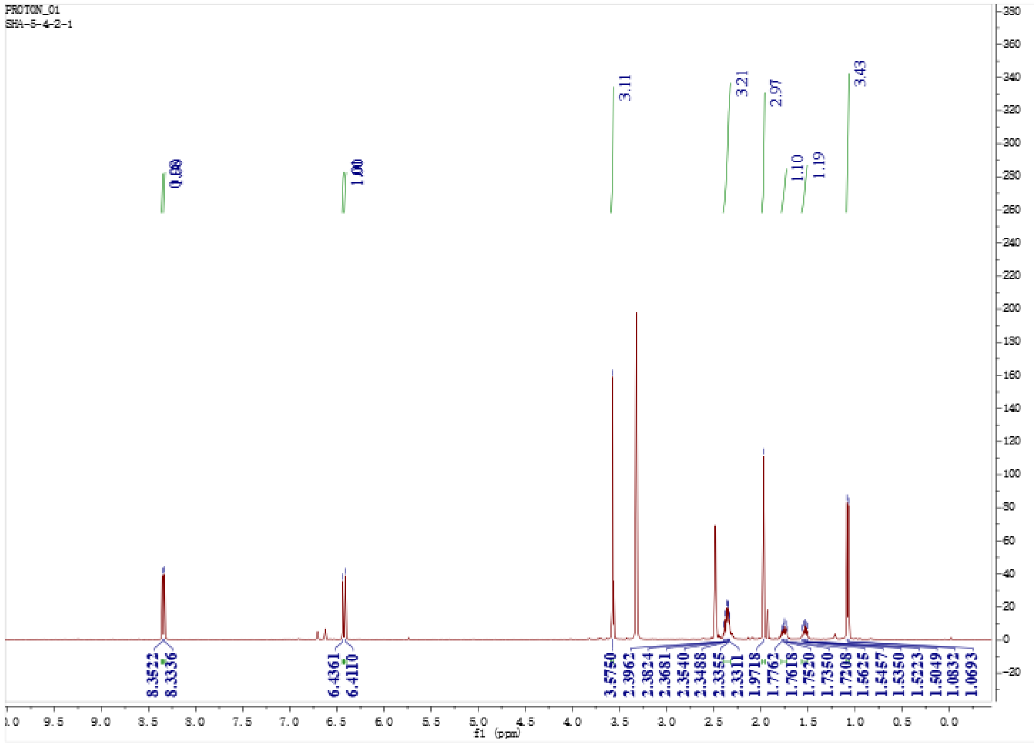

Figure S23. ^{13}C NMR (125 MHz, DMSO-d_6) spectrum of compound **3**.

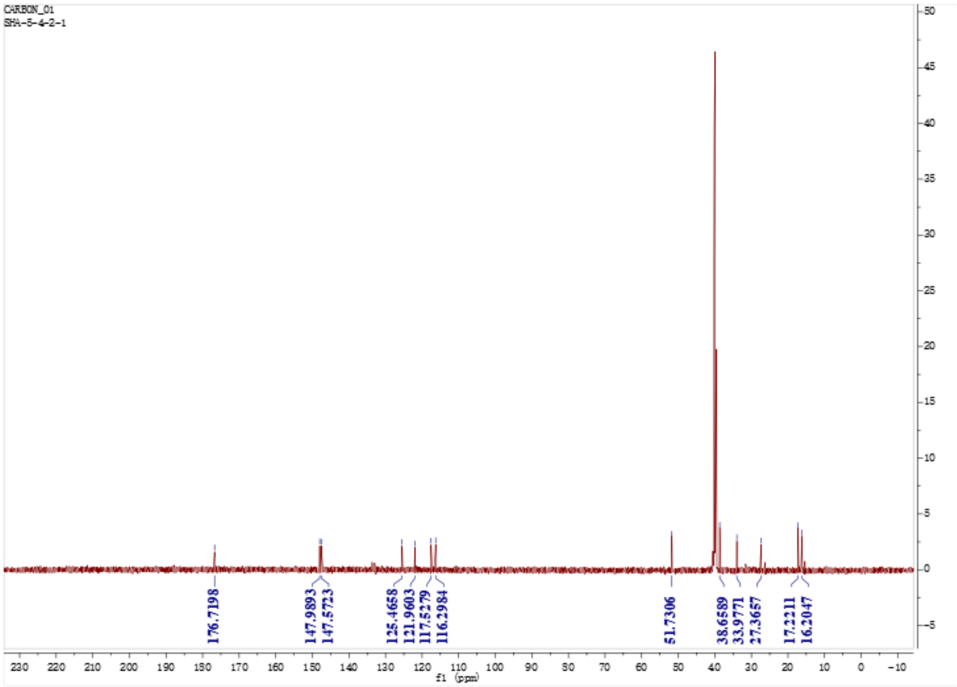

Figure S24. DEPT (125 MHz, DMSO-d_6) spectrum of compound **3**.

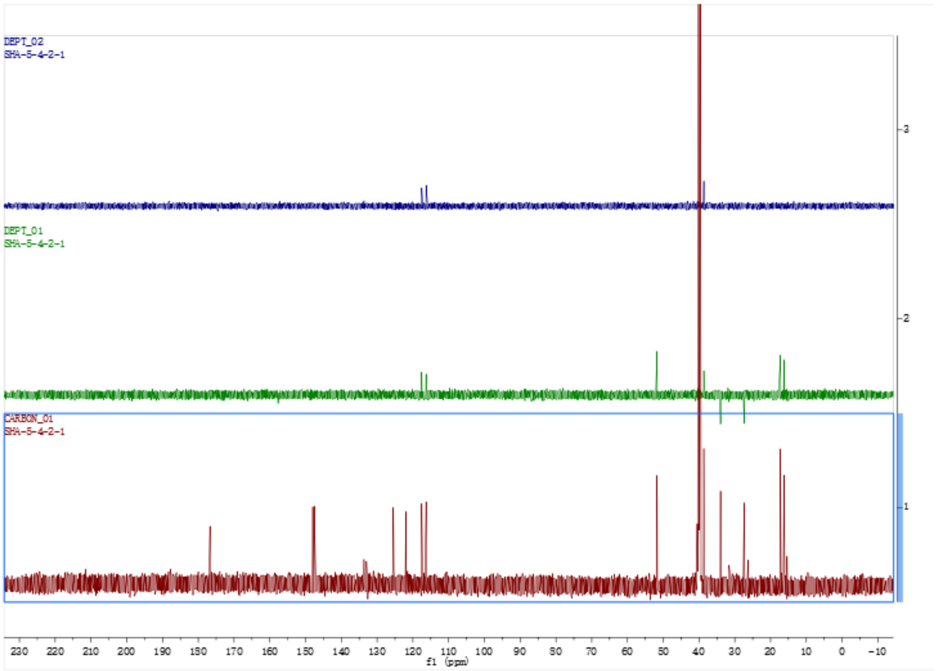

Figure S25. ^{1}H-^{1}H COSY spectrum of compound **3** in DMSO-d_6.

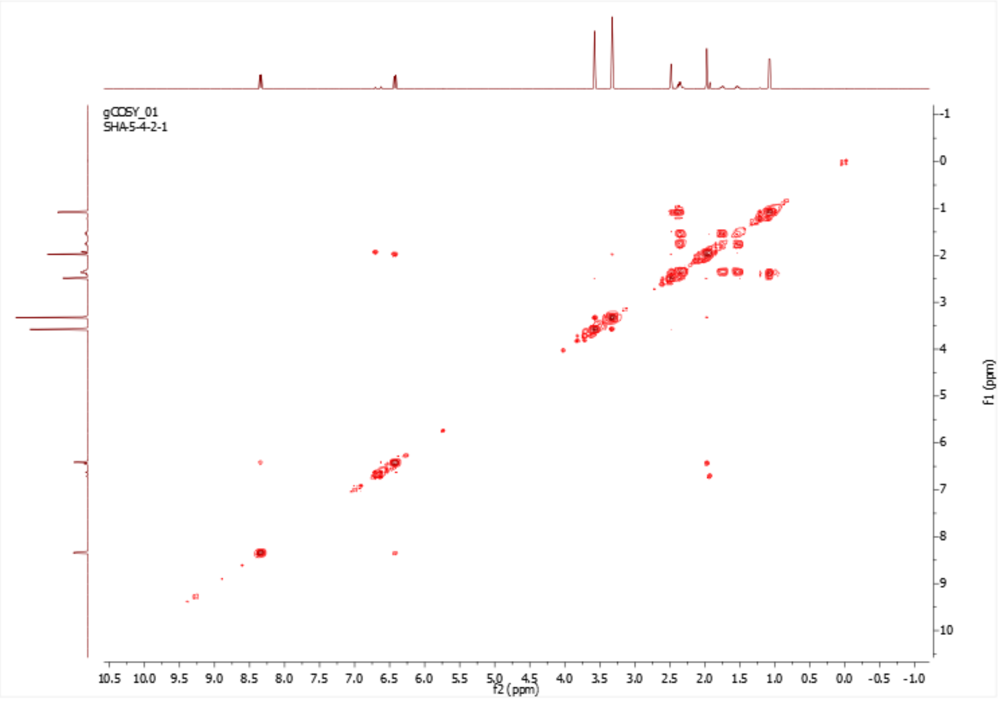

Figure S26. HSQC spectrum of compound **3** in DMSO-d_6.

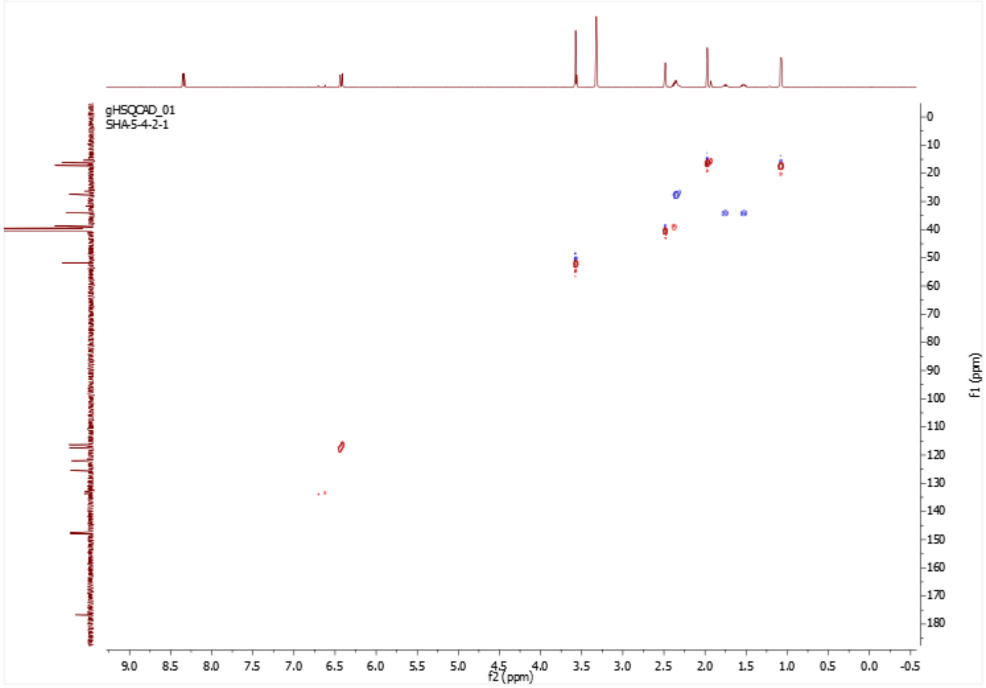

Figure S27. ^{1}H-^{13}C HMBC spectrum of compound **3** in DMSO-d_6.

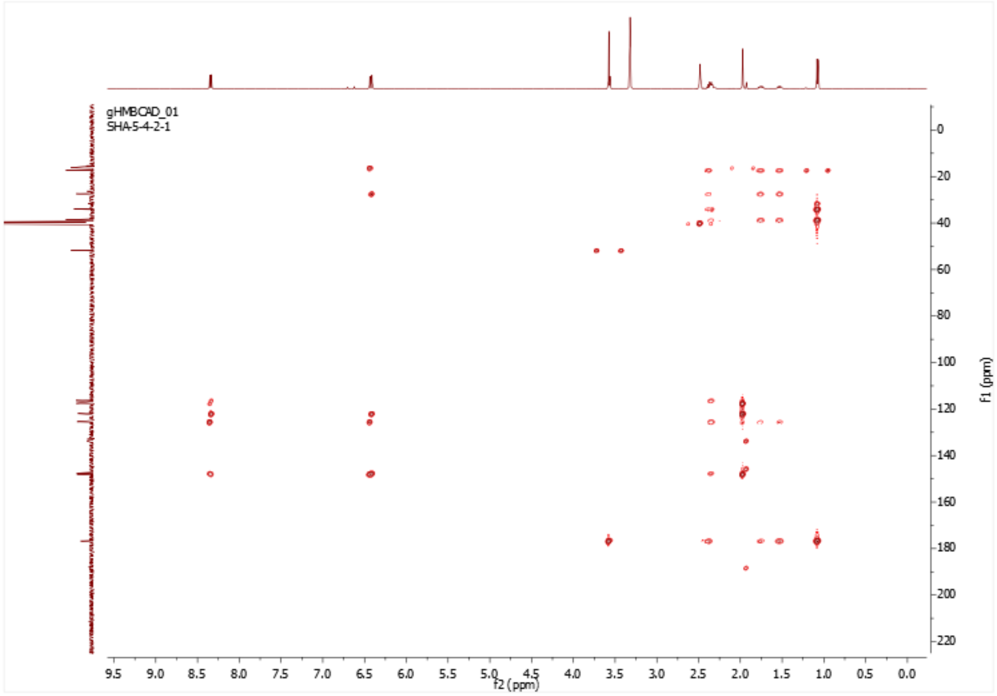

Figure S28. NOESY spectrum of compound **3** in DMSO-d_6.

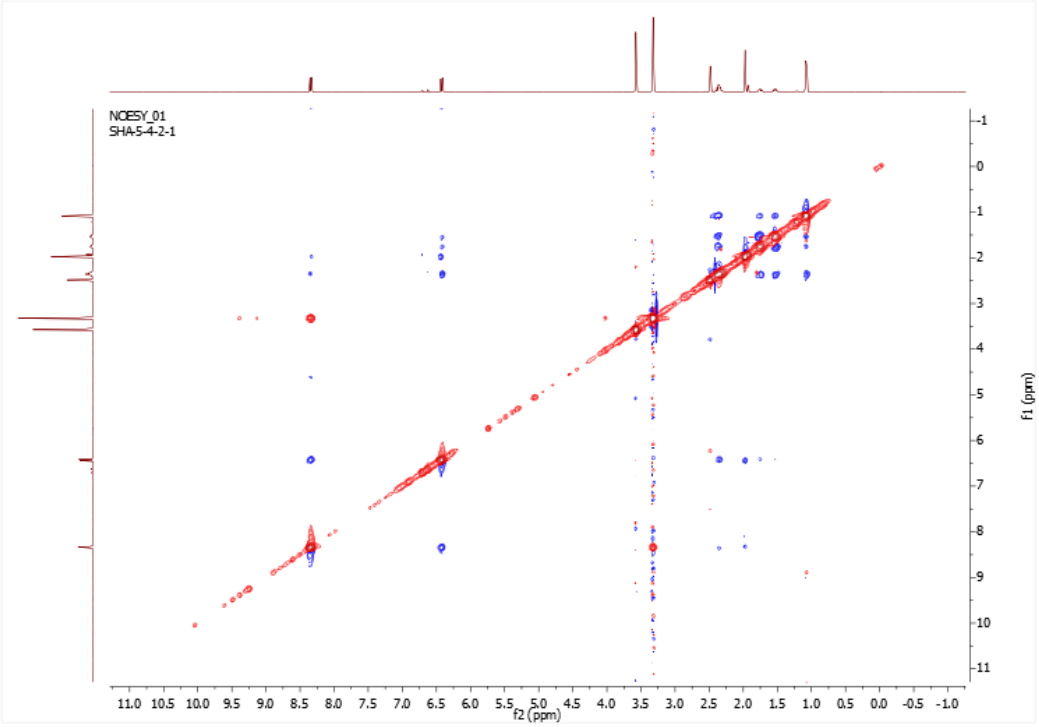

Figure S29. HRESIMS spectrum of compound **3**.

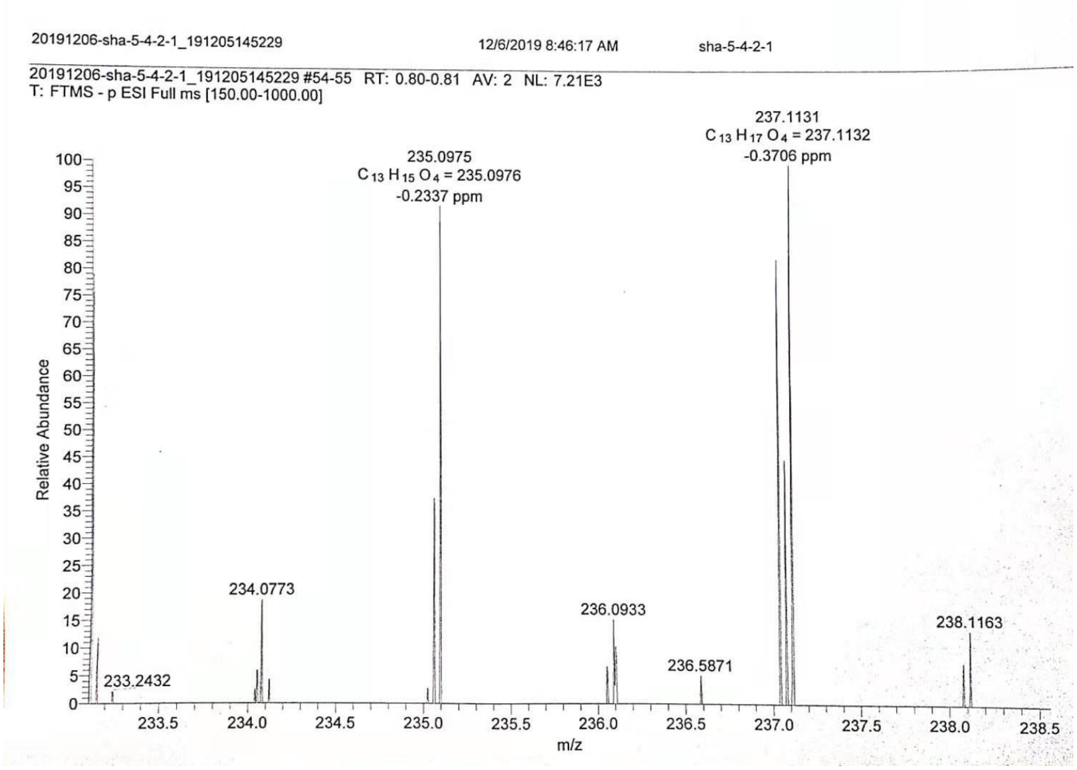

Figure S30. IR spectrum of compound **3**.

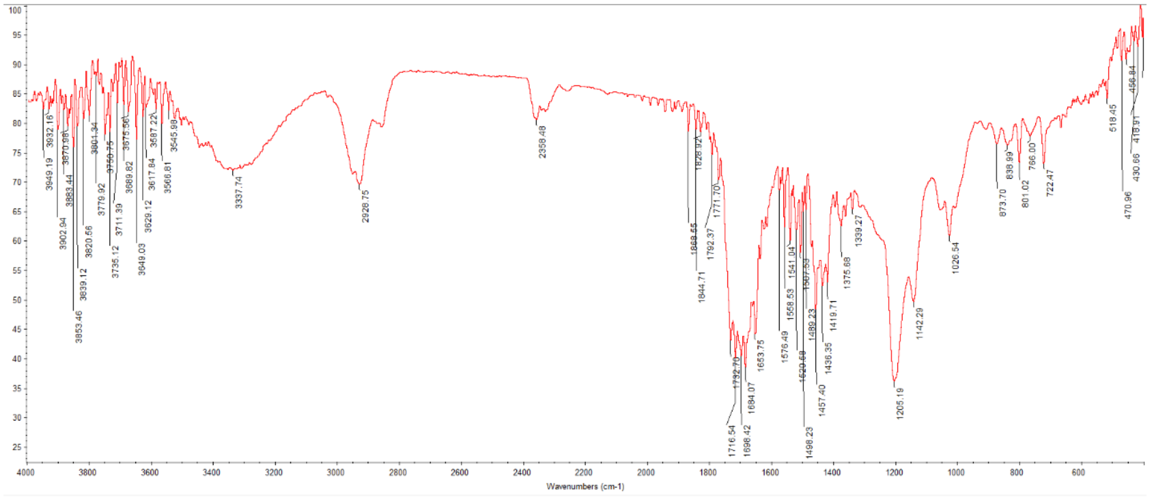

Figure S31. UV spectrum of compound **3**.

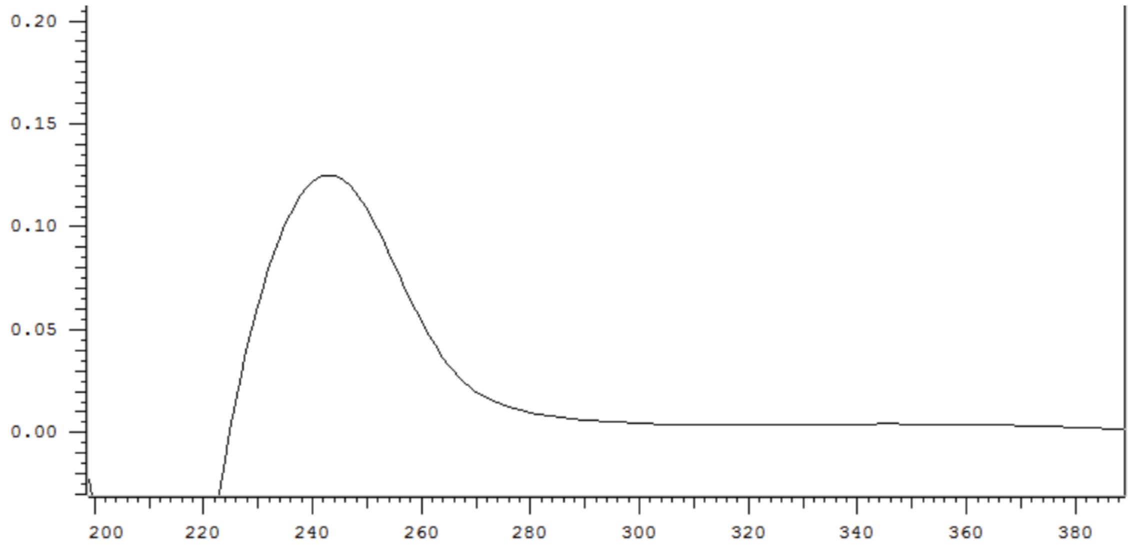

Figure S32. ^{1}H NMR (500 MHz, $CDCl_3$) spectrum of compound **1**.

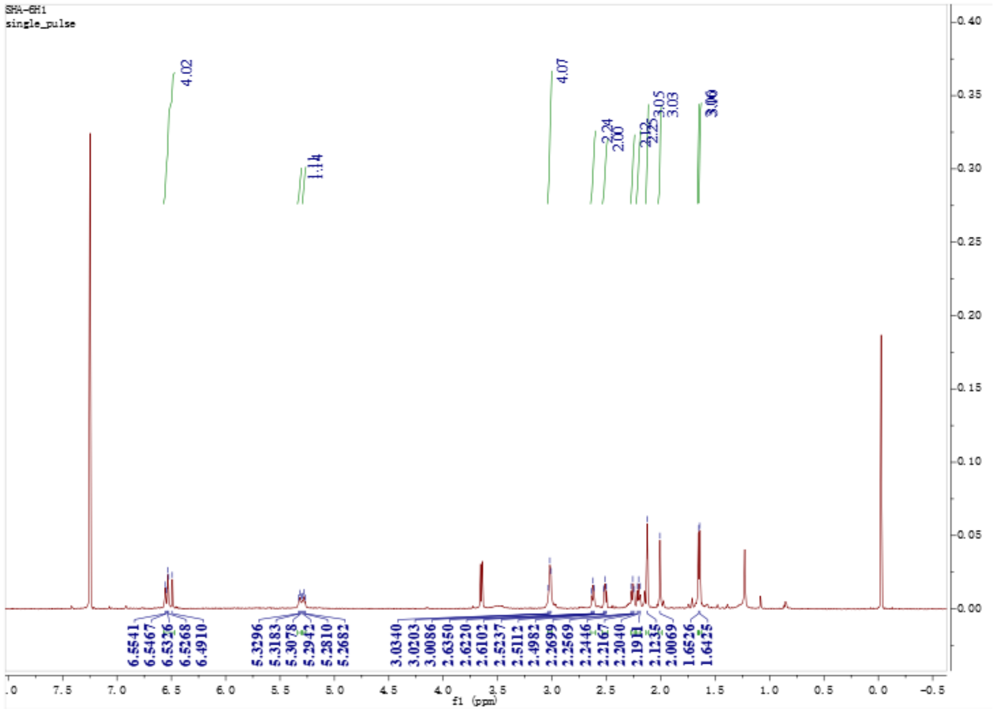

Figure S33. ^{13}C NMR (125 MHz, $CDCl_3$) spectrum of compound **1**.

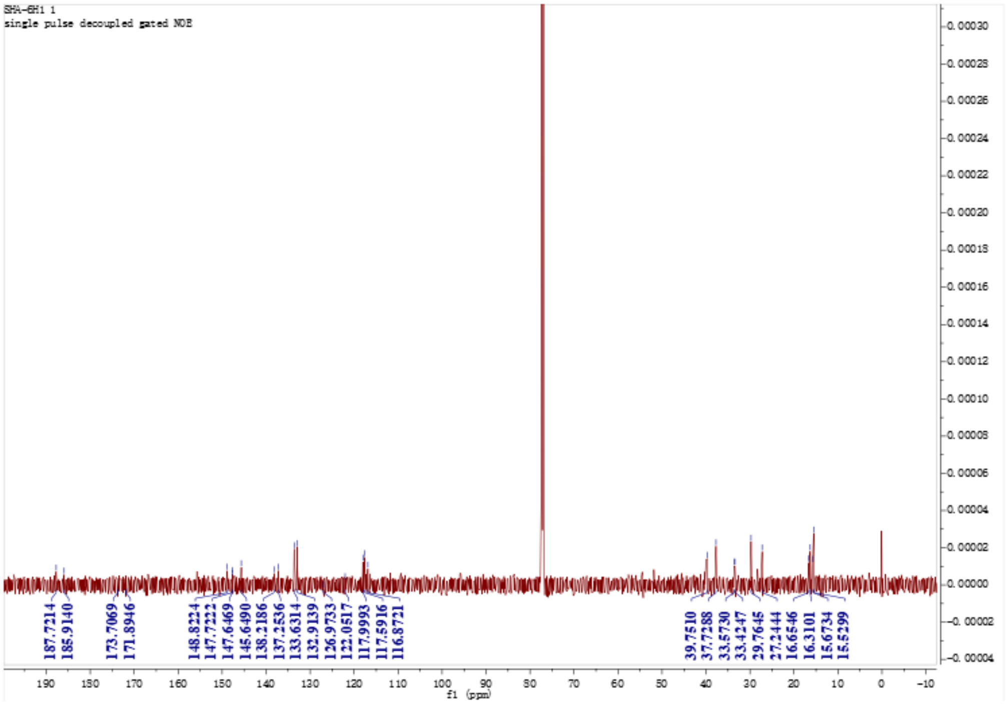

Figure S34. DEPT (125 MHz, $CDCl_3$) spectrum of compound **1**.

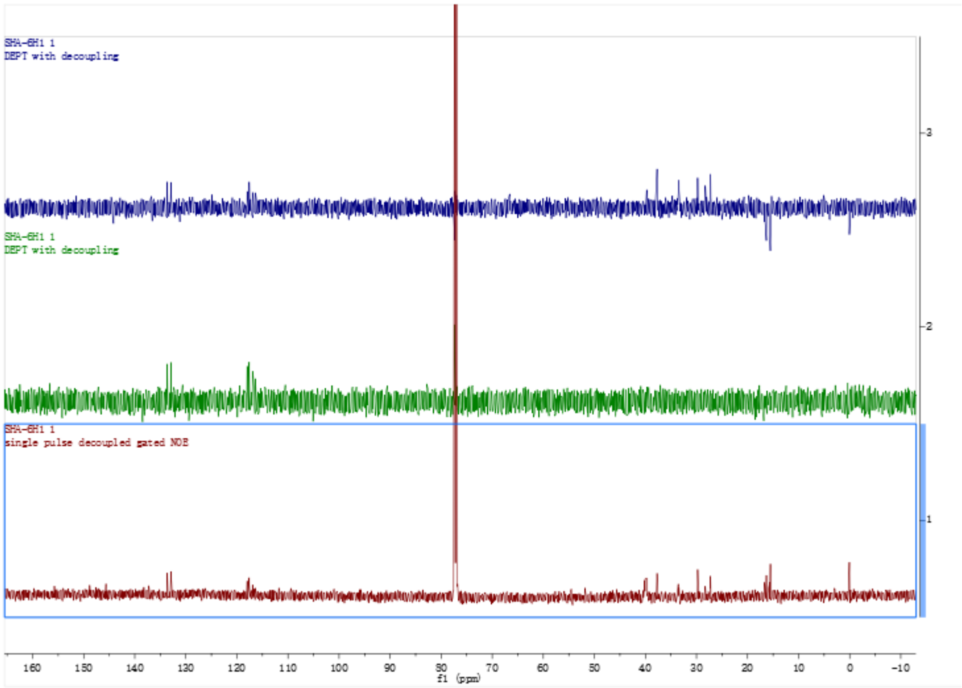

Figure S35. ^{1}H-^{1}H COSY spectrum of compound **1** in $CDCl_3$.

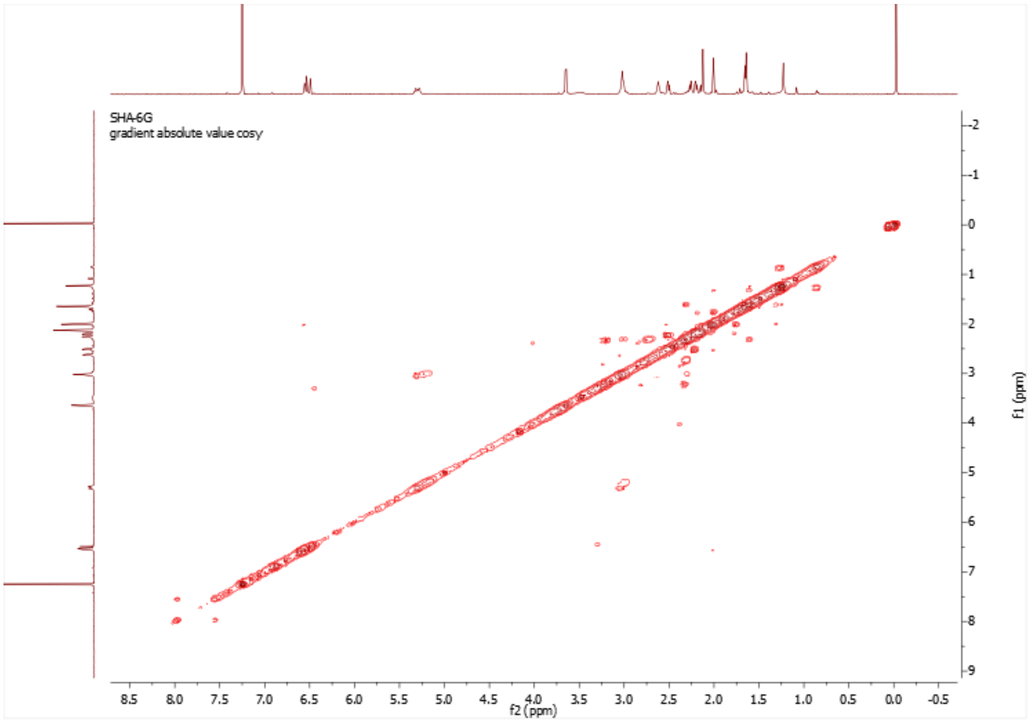

Figure S36. ^{1}H-^{13}C HSQC spectrum of compound **1** in $CDCl_3$.

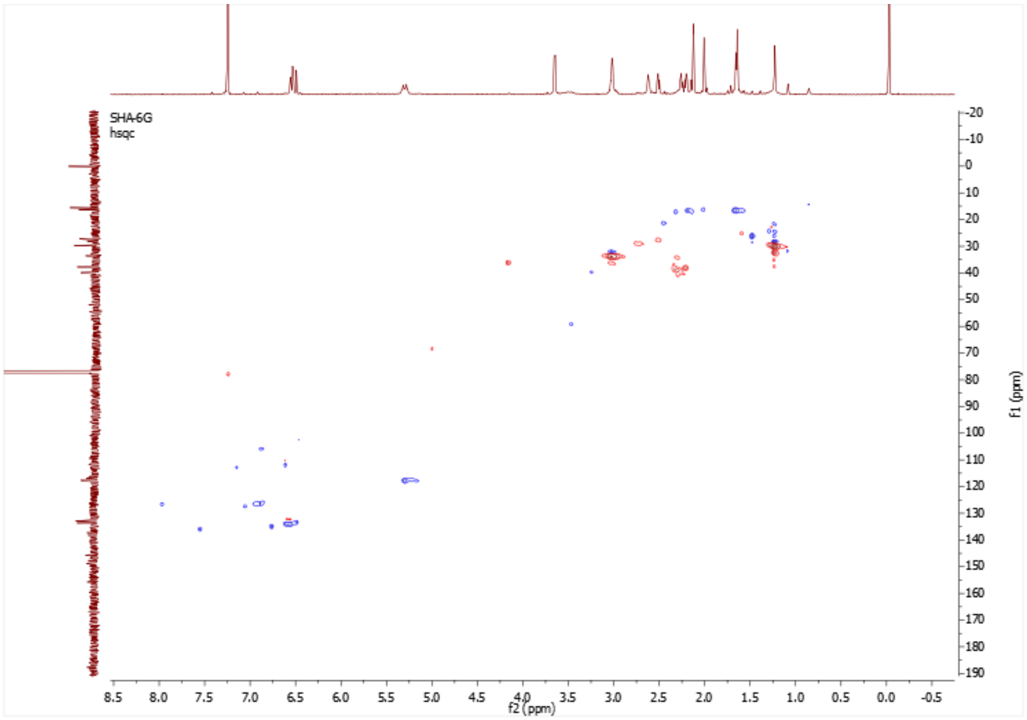

Figure S37. ^{1}H-^{13}C HMBC spectrum of compound **1** in $CDCl_3$.

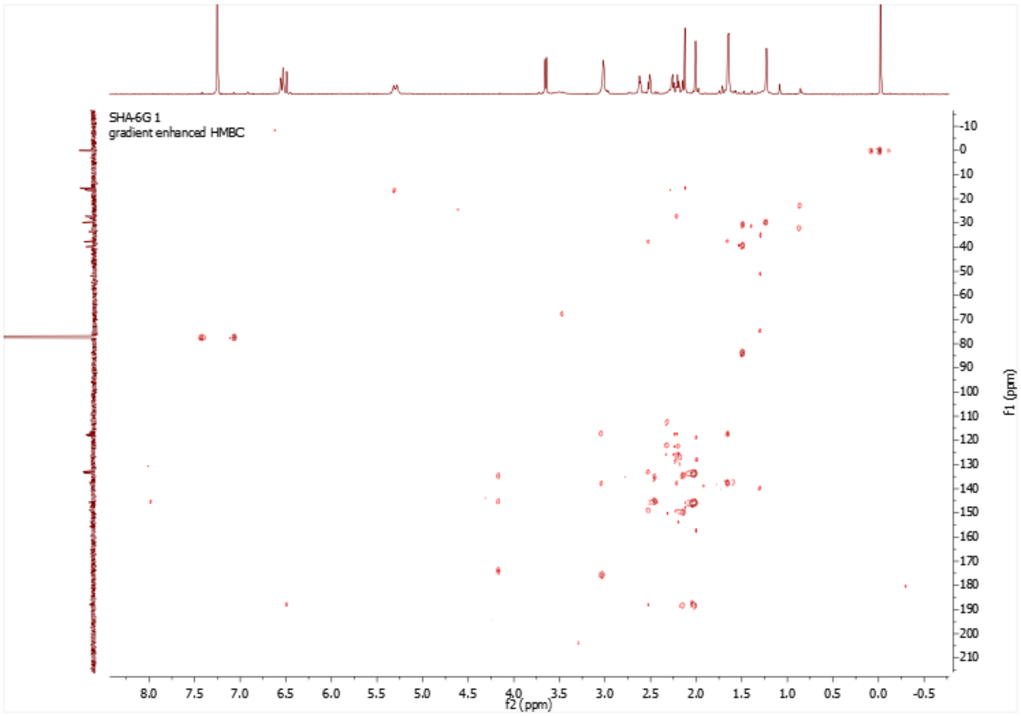

Figure S38. NOESY spectrum of compound **1** in $CDCl_3$.

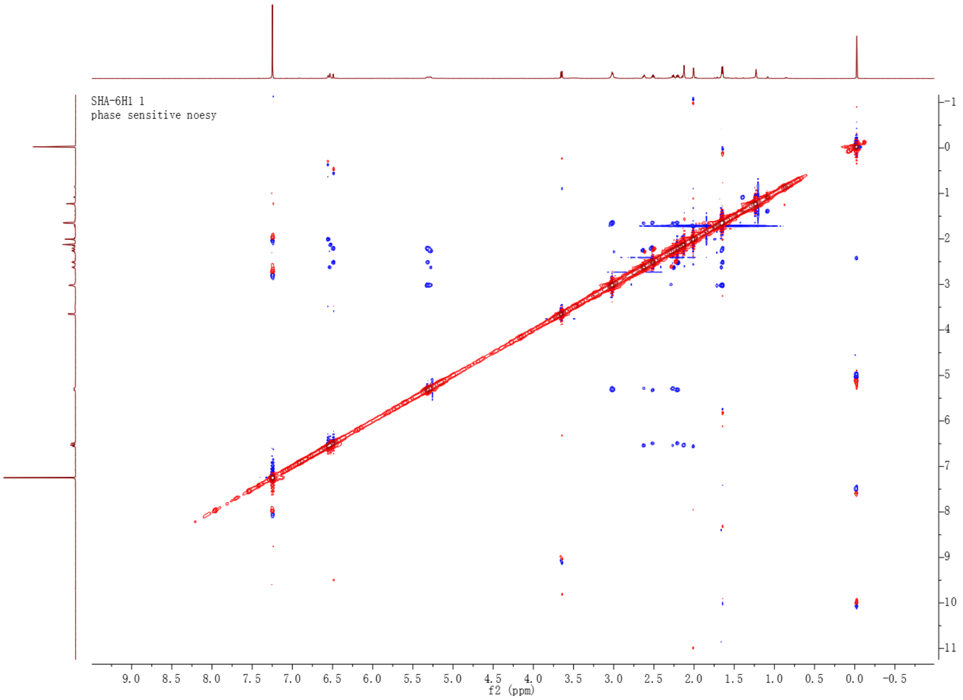

Figure S39. ^{1}H NMR (500 MHz, $CDCl_3$) spectrum of compound 2.

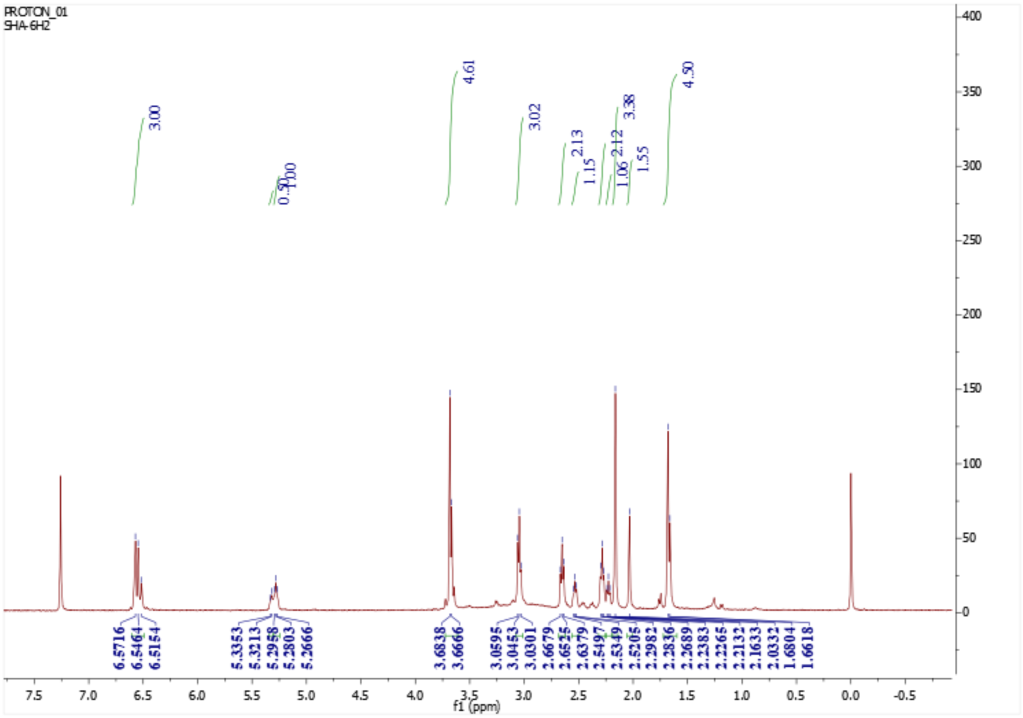

Figure S40. ^{13}C NMR (125 MHz, $CDCl_3$) spectrum of compound **2**.

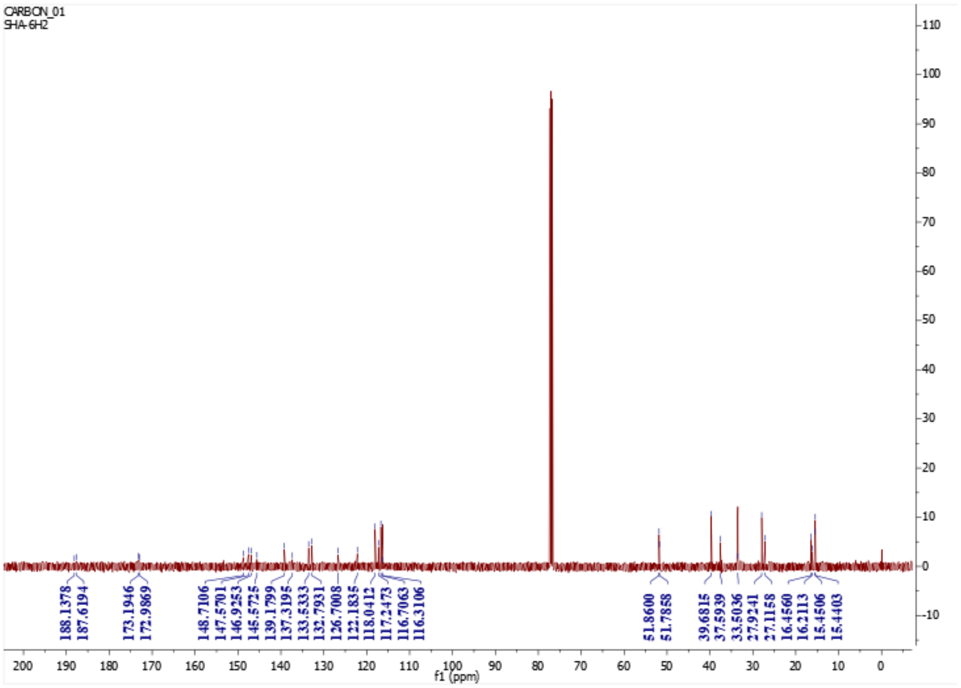

Figure S41. DEPT (125 MHz, $CDCl_3$) spectrum of compound **2**.

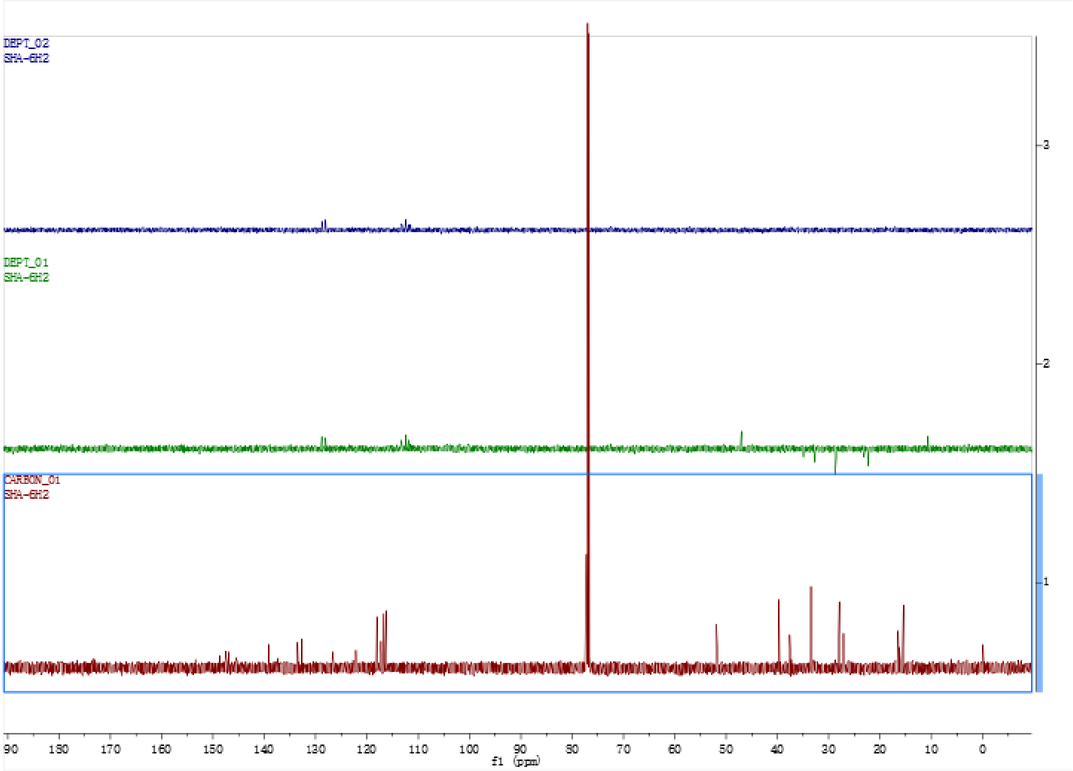

Figure S42. ^1H-^1H COSY spectrum of compound **2** in $CDCl_3$.

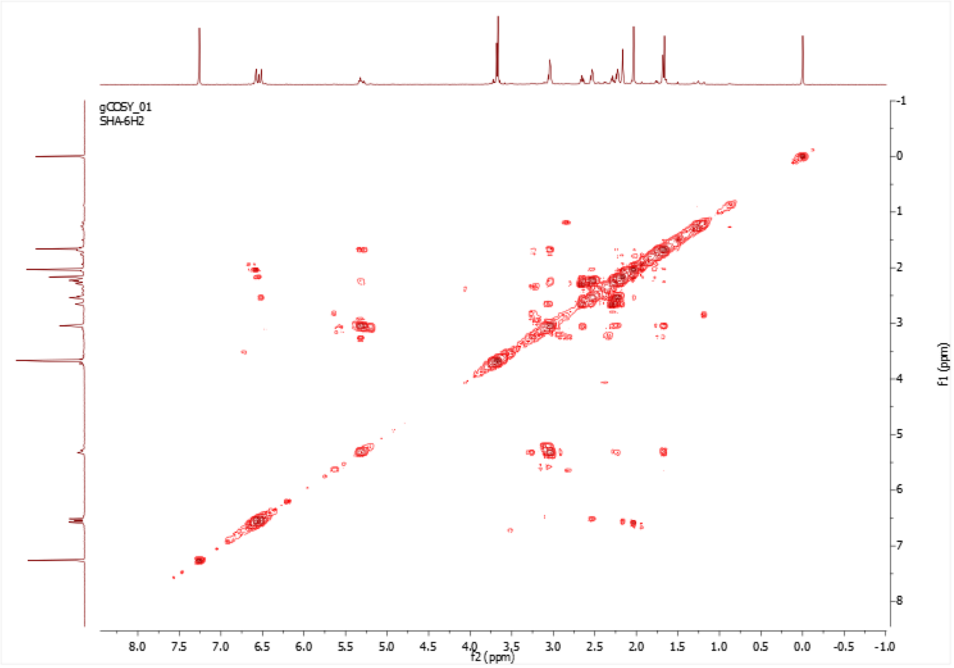

Figure S43. HSQC spectrum of compound **2** in $CDCl_3$.

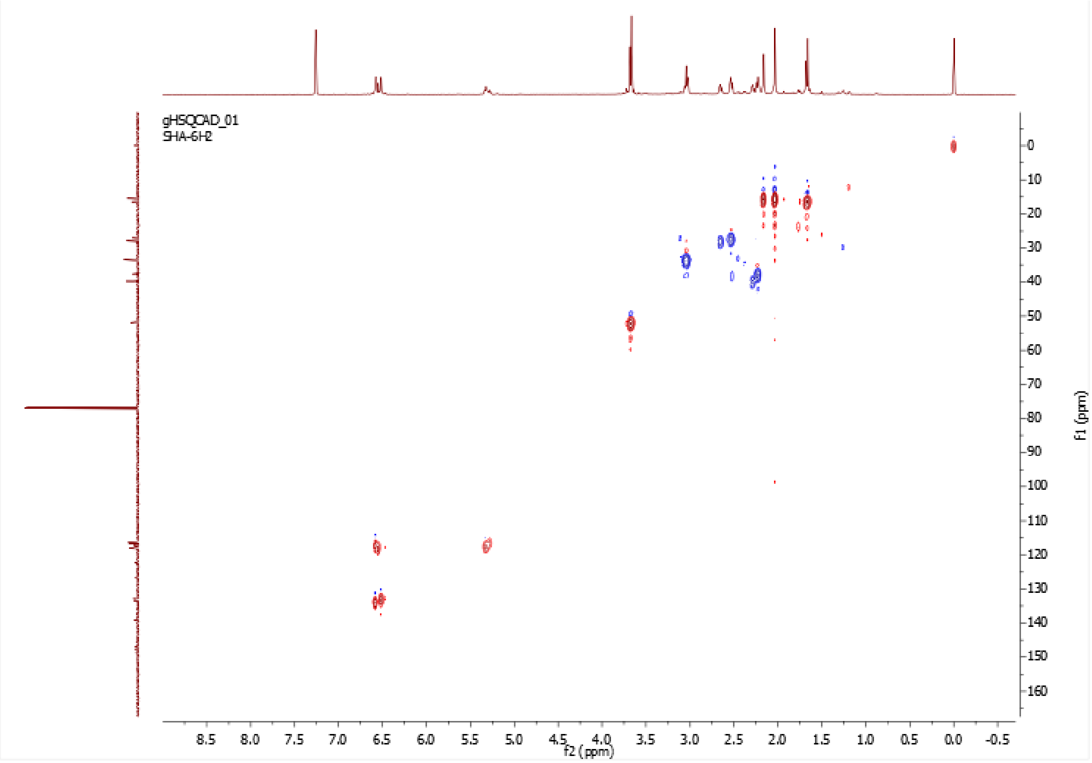

Figure S44. ^{1}H-^{13}C HMBC spectrum of compound **2** in $CDCl_3$.

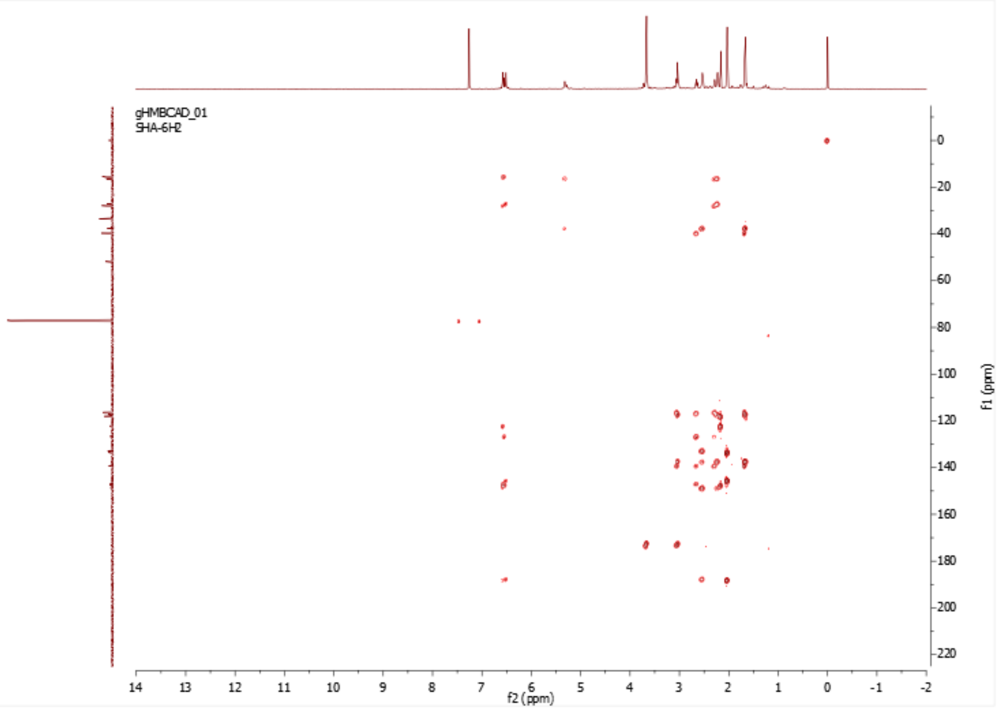

Figure S45. NOESY spectrum of compound **2** in $CDCl_3$.

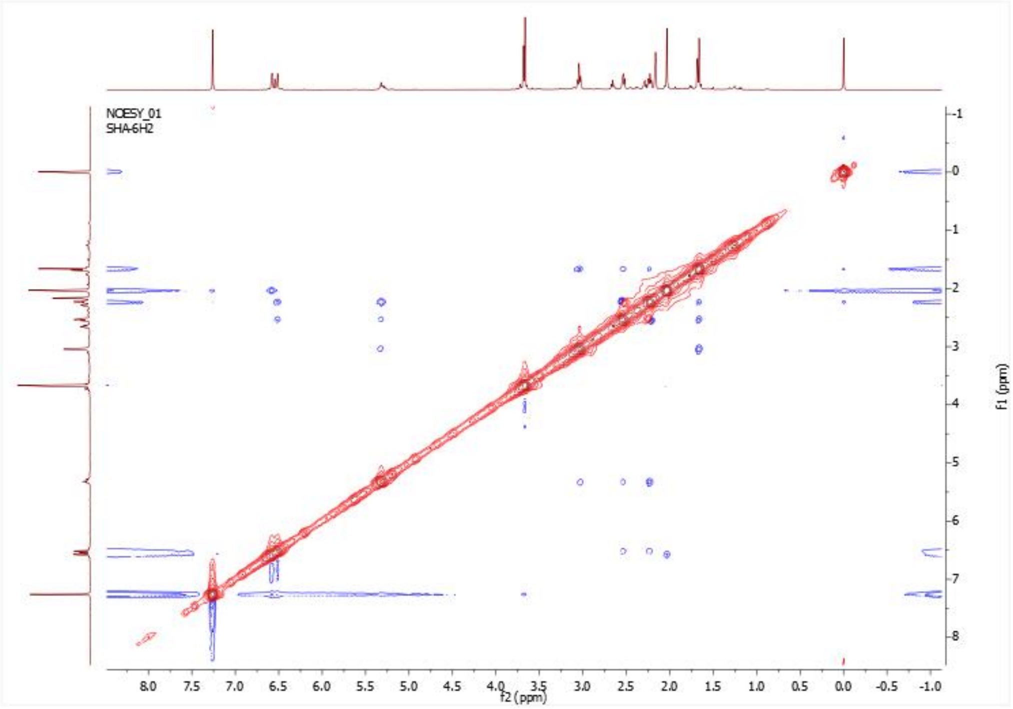

Figure S46. ^{1}H NMR (500 MHz, $CDCl_3$) spectrum of compound **3**.

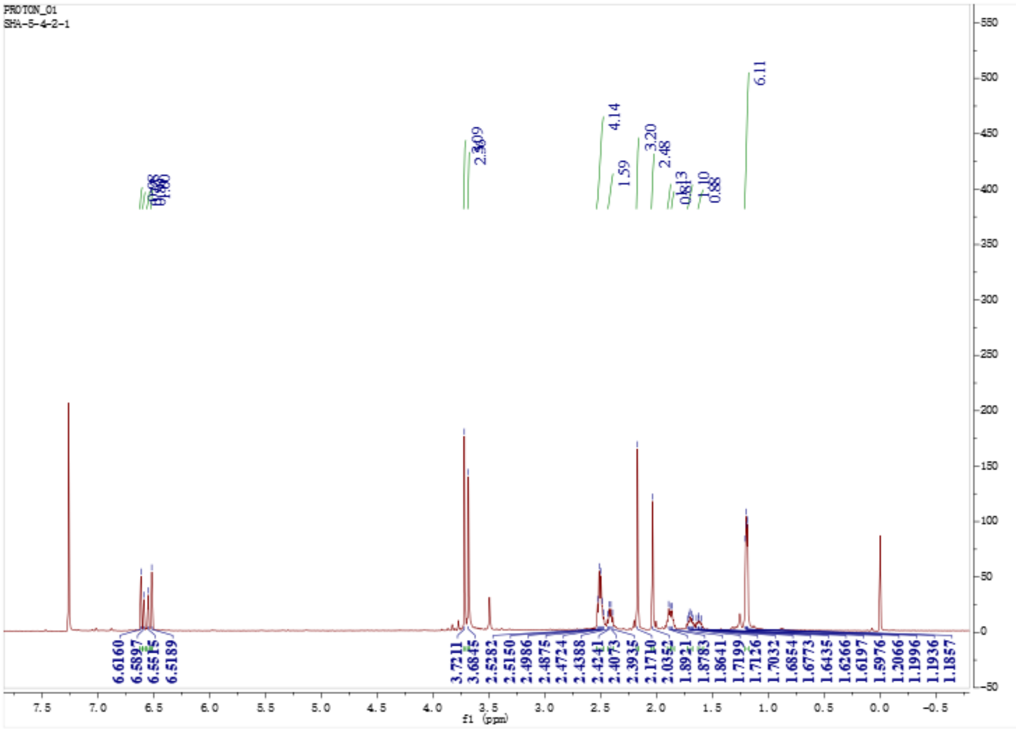

Figure S47. ^{13}C NMR (125 MHz, $CDCl_3$) spectrum of compound **3**.

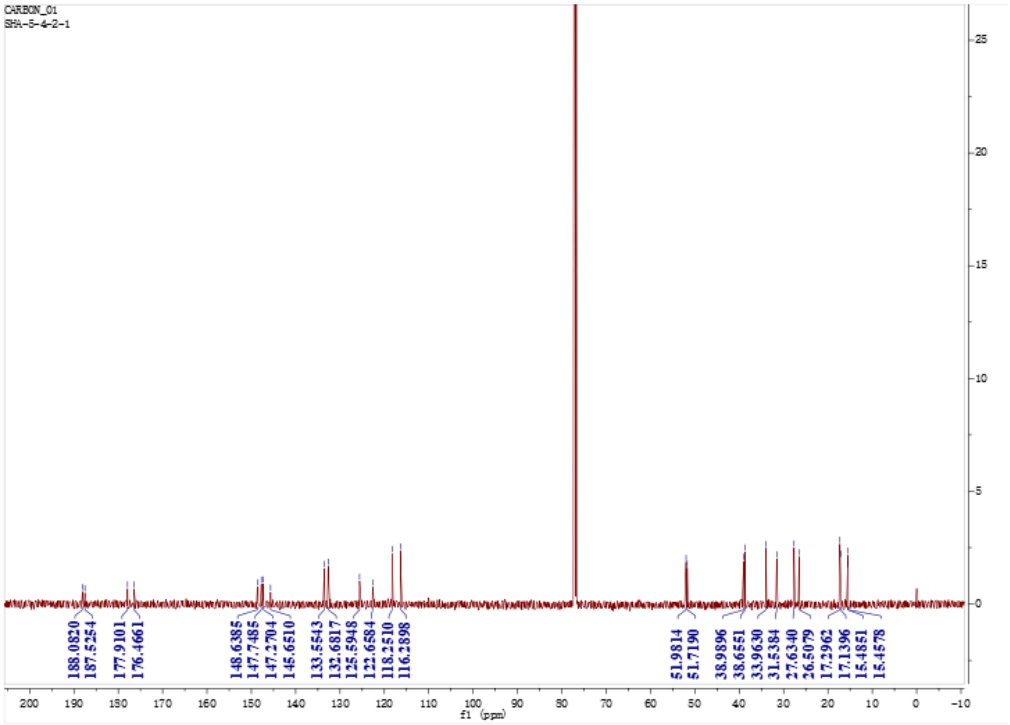

Figure S48. DEPT (125 MHz, $CDCl_3$) spectrum of compound **3**.

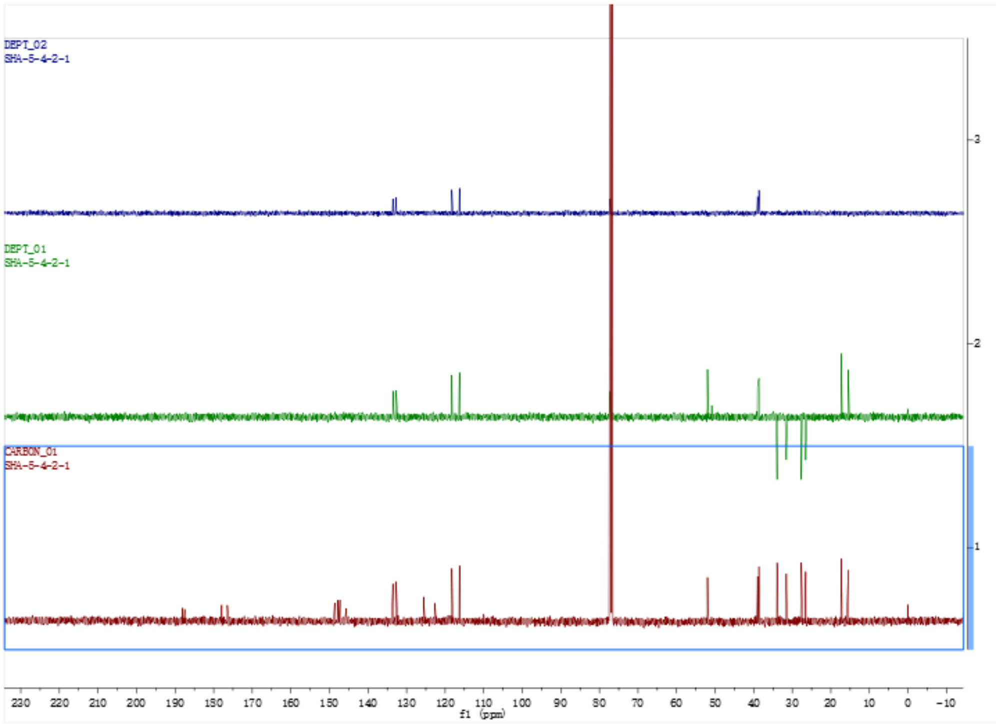

Figure S49. ^{1}H-^{1}H COSY spectrum of compound **3** in $CDCl_3$.

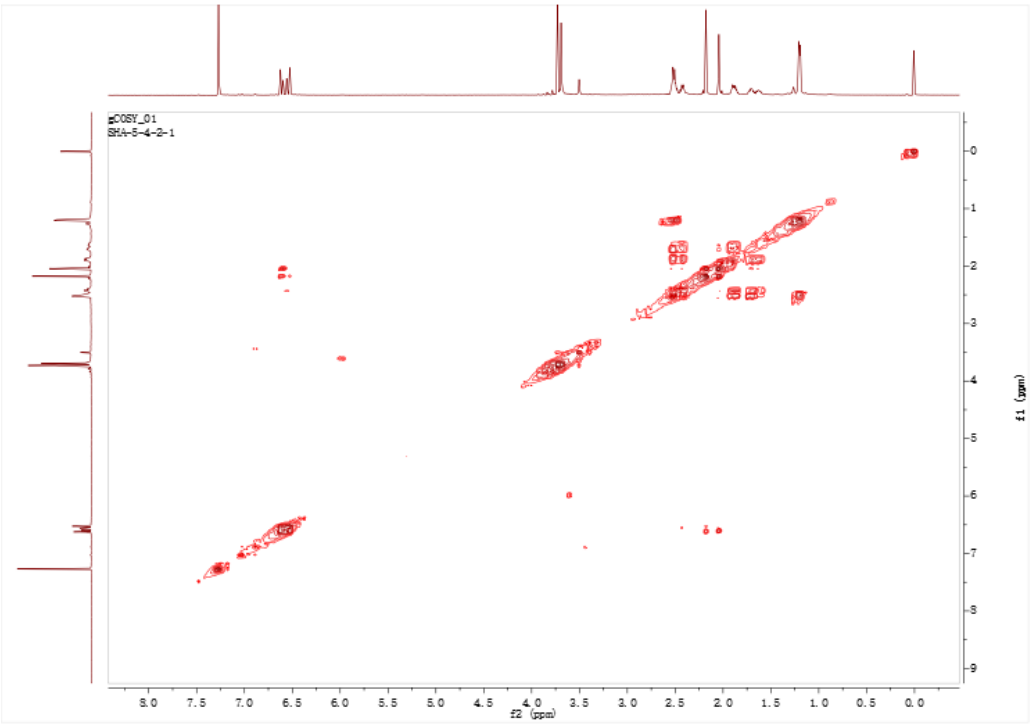

Figure S50. HSQC spectrum of compound **3** in $CDCl_3$.

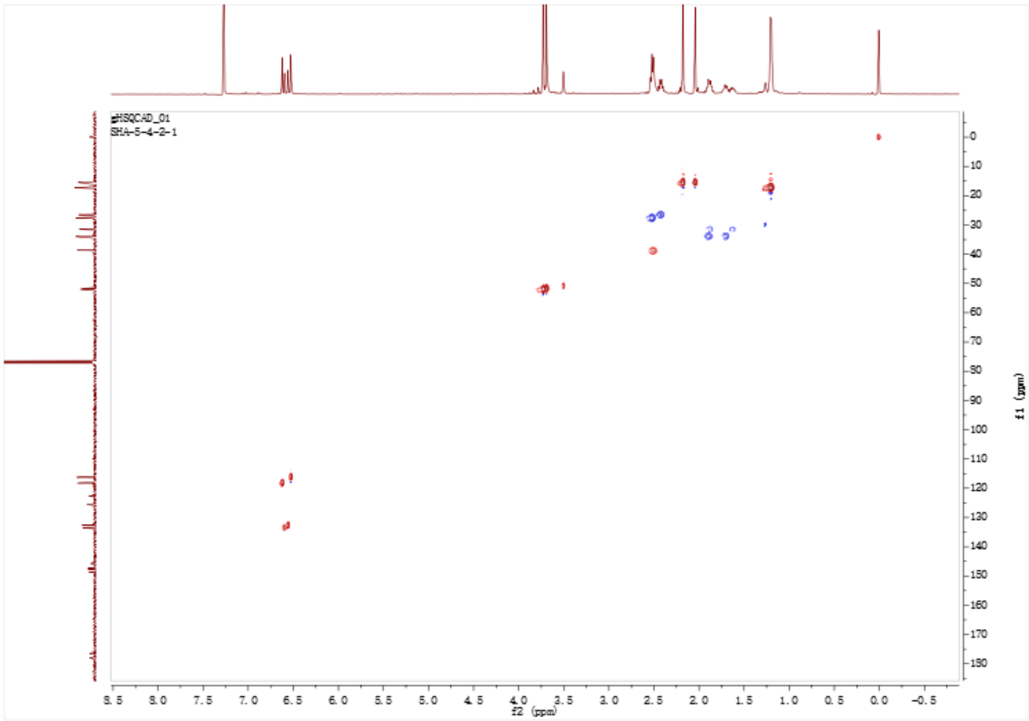

Figure S51. ^{1}H-^{13}C HMBC spectrum of compound **3** in $CDCl_3$.

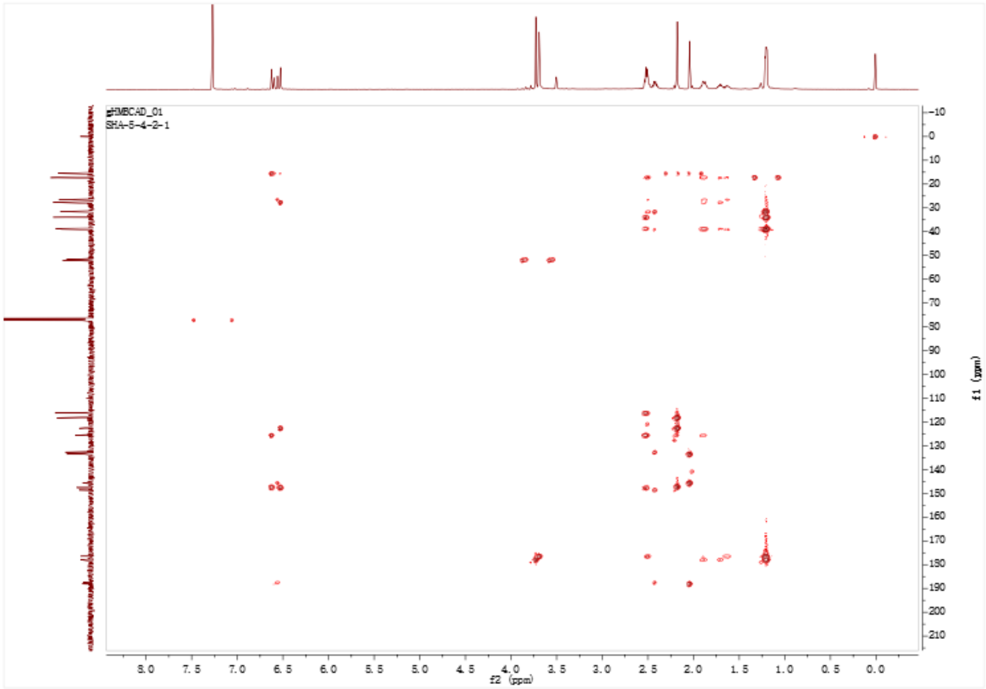

Supplement: Supplementary file 1 [file marinedrugs-18-00071-s001.pdf]
